# White light emitting diode based on purely organic fluorescent to modern thermally activated delayed fluorescence (TADF) and perovskite materials

**DOI:** 10.1186/s40580-019-0201-6

**Published:** 2019-09-16

**Authors:** Dipjyoti Das, Peddaboodi Gopikrishna, Debasish Barman, Ramesh Babu Yathirajula, Parameswar Krishnan Iyer

**Affiliations:** 1 Center for Nanotechnology, Indian Institute of Technology Guwahati, Guwahati, Assam 781039 India; 2 Department of Chemistry, Indian Institute of Technology Guwahati, Guwahati, Assam 781039 India

**Keywords:** Solution processed white light emitting diodes, Organic light emitting diodes, Polymer light emitting diodes, Thermally activated delayed fluorescence, Perovskite light emitting diodes

## Abstract

White organic/polymer light emitting diode (WOLED/WPLED) processed from solution has attracted significant research interest in recent years due to their low device production cost, device flexibility, easy fabrication over large area including roll to roll and ability to print in various designs and shapes providing enormous design possibilities. Although WOLEDs fabricated using solution process lack their thermally evaporated counterparts in terms of device efficiency, remarkable progress has been made in this regard in recent years by utilizing new materials and device structures. In the present review, we have summarized and extrapolated an excellent association of old and modern concept of cost-effective materials and device structure for realization of white light. In particular, this article demonstrated and focused on design, and development of novel synthesis strategy, mechanistic insights and device engineering for solution process low cost WOLEDs device. Herein, an overview of the prevailing routes towards white light emitting devices (WLEDs) and corresponding materials used, including polymer based WLED, small molecules emitters based thermally activated delayed fluorescence (TADF), perovskite light-emitting diodes (PeLEDs) and hybrid materials based LEDs, color down-converting coatings with corresponding best efficiencies ever realized. We presume that this exhaustive review on WLEDs will offer a broad overview of the latest developments on white SSL and stonework the approach en route for innovations in the immediate future.

## Introduction

As the world gets modernized, electronic devices have become an inevitable part of our daily lives and with increasing dependence of artificial intelligence and machine learning a life without electronic gadgets would be impossible to imagine. Our dependability on electronics has stretched to such an extent that our lifestyle, economic activity, heath as well as security rely and are impacted hugely by development in the electronic technology. As a result, there has been an ever-increasing demand of highly efficient, eco-friendly, flexible and low cost integrated optoelectronic devices and the resources and methodologies used to manufacture such devices has become a subject of extreme importance. Considering the unique and easy to tune chemical and optical properties, flexibility and stretchablity of the organic materials, which cannot be afforded by the conventional materials in use, these organic electronic devices are being viewed as the future of electronics, thereby expanding the functionality and accessibility of electronics [1–3]. Organic electronic devices have the potential to be more energy efficient and eco-friendly, can be processed and manufactured using more resource friendly and cost-effective processes, and most importantly can be flexible and stretchable unfolding vast design possibilities. The prime focus of the researchers working in the field of organic electronics is mainly on displays and lighting, transistors and solar cells, healthcare and biomedical devices, both in terms of designing novel materials as well as device architectures. In recent years significant research interest has been devoted towards various organic optoelectronic devices such as OLEDs, OSCs, OFETs and organic memories etc [4–8]. Especially, OLEDs that can emit white light, is considered to be the next generation lighting source due to the limitations of the traditional incandescent bulbs in terms of device efficiency, the non-eco-friendly nature of the fluorescence tubes and the restriction in the fabrication of large area light source by using inorganic semiconductors [9–12]. However, in order to become the next generation lighting source by replacing the traditional lighting elements, WOLEDs should be able to demonstrate higher efficiency maintaining a reduced production cost. Unfortunately, most of the highly efficient WOLEDs have been fabricated via thermal deposition technique at high vacuum level giving rise to noteworthy challenges towards manufacturing low cost large area devices. The lookouts for new processing techniques are hence increasing in order to make large area electronics available for general applications at a reduced cost. In that regard, solution based device processing procedures, viz. spin-coating, ink-jet printing etc. offer numerous advantages over the existing manufacturing process such as vacuum deposition. Apart from low cost, solution processed OLEDs also hold an edge over other techniques since it allows printing in various designs and shapes and provides enormous design possibilities. Due to their ability to form high quality thin films through solution processing technique, polymer materials have often been the preferred material while fabricating solution processed OLEDs. Polymer based WOLEDs (WPLEDs) can be easily fabricated using the aforementioned solution processed techniques reducing the production cost significantly. From materials point of view, the relatively easy color tuning property of the π-conjugated polymers as well as the easy to control doping process leads to the availability of a large variety of materials to be used as the active material for WPLEDs. Apart from utilization of polymers as the emitting layer and host materials, significant research effort has also been devoted to design and develop solution processed small molecule based white light materials. Another important aspect towards the realization of high-performance white light is the utilization of triplet excitons in the radiative recombination process. Materials containing heavy atoms such as Ir, Pt etc. have the capability to induce spin orbit coupling thereby allowing the radiative decay of triplet excitons. Although WOLEDs based on such materials exhibits higher efficiency, they are not cost effective. Recently, Adachi and co-workers pioneered a 100% excitons utilization strategy in OLEDs via TADF emitters exploiting purely organic materials, which, under thermal activation, could harvest both 25% as well as 75% of singlet triplet excitons respectively via reverse intersystem crossing (RISC) from their lowest triplet excited state (T1) to the lowest singlet excited state (S1), facilitated by the narrow energy gap between S1 and T1 [13]. These TADF based emitters are therefore capable of demonstrating 100% IQE with an EL efficiency on par with that of the Ph-OLED. Moreover, tremendous work has been done recently to generate WLED by adopting TADF emitters, due to their higher triplet in energy and high full width at half maxima (FWHM), that enables to transfer full energy either to counter the green and red fluorescent emitter or by complementary yellow emitter [14, 15]. To suppress the carrier recombination in the device, TADF emitters are being explored as assistant dopant and host, through Förster resonance energy transfer (FRET) or exciplex formation mechanism. Remarkably, low cost solution processed TADF-WOLEDs have been fabricated by strategically employing single and multi-layer TADF emissive layer, realizing very low turn on voltages (V), high power efficiencies (PE (lm/W)), current efficiencies (CE (cd/A)) even maximum of 28% external quantum efficiencies (ηEQE) with Commission Internationale de L’Eclairage (CIE) co-ordinate near to standard (0.33, 0.33) extending towards a promising lighting technology for future generation WOLEDs. Recently, perovskite-based materials have emerged as probable contenders for high performance devices. Perovskite materials play a vital role in the field of optoelectronics and photovoltaics, due to their high crystallinity, higher mobilities, good film forming properties, carrier longer life times as well as higher quantum yields. The beauty of perovskite materials is that, by performing simple band gap tuning and adding additives like long chain cations, amines, bromides, one can tune the color of light as well as improve the device parameters to be able to be used in multiple applications. Perovskite metal halides have already been utilized to demonstrate excellent efficiency in solar cells and significant amount of research have now been dedicated towards their utilization in LED devices by exploring different means of tuning their optoelectronic and luminescence properties. This has led to a plethora of highly photoluminescent new materials (including solution processed), covering the entire visible range, associated with remarkable improvement in device performances.

Overall, WOLEDs have the potential to fulfil the future demands such as energy saving, foldable, high color quality, high brightness and most importantly large area display and solid-state lighting devices. Although, there are still challenges to be overcome, advanced WOLED systems can also be useful to develop stretchable and transparent displays. The present commercialized WOLED products are manufactured by thermal deposition techniques, which is more expensive. Therefore, there is enough scope of research to develop highly efficient WOLEDs by solution process technique by proper designing of novel materials and device architectures.

Some of the major issues, solution processed WOLEDs are facing is long-term device stability and chemical modification (in case of perovskite materials). The device stability mainly depends on the active material. It is well-known that the organic materials are more stable as compared to perovskite materials. Hence, these problems can be solved by chemical or physical doping of organic materials into the perovskite materials. Another important issue is the color stability of the WOLEDs. It is quite difficult to obtain pure white light from the device over a wide voltage range. This problem can be solved by focusing on single white light emitting materials.

In this review, a systematic description has been provided regarding the working of an OLED, approaches to generate white light, important characterization parameters and finally solution processed WOLEDs fabricated using π-conjugated materials, especially the polymers have been discussed and reviewed, to be inclusive of literature in the best possible manner. Special emphasis has also been given to highlight the recent development of high performance TADF WOLEDs by employing some π-conjugated unique small molecules with specific device engineering strategy to produce white emission. Lastly but not the least, the recent developments in the emerging area of PeLEDs, both for material synthesis and device architecture, and towards their realization of white light has also been discussed. We expect this review to be helpful for the reader in providing them an overview of the past and present state towards the development of cost-effective materials and novel device architecture of white organic light emitting diode using solution processing technique and possible direction to overcome the existing challenges for developing futuristic solid state lighting (SSL) materials and devices.

## Basics of OLED and characterization parameters

As the name suggests, in an OLED, light is generated in an organic semiconductor layer known as emissive layer. In an OLED, usually one or more organic thin films is traditionally sandwiched as an intermediate layer with two distinct electrodes at top and bottom where one electrode is usually transparent in order for light to escape from the device. Upon electrical excitation, electrons get injected from the cathode side while the holes are injected from the anode. As they meet and recombine at the EML, they give light as output. The color of the light generated by the OLEDs is often determined by the property of the emissive materials. The role of the others layers used in the OLED structure is mainly to provide efficient carrier injection as well as confining them in the EML to achieve more recombination and higher efficiency. The performance of a WPLED is usually judged by both device efficiency and color quality. In case of efficiency, three different terms have been used widely viz. luminous efficiency, quantum efficiency and power efficiency. Among the three parameters, the first two are material dependent and therefore important for material evaluation while the third one can be related to the device architecture and is therefore crucial in device characterization and fabrication improvements. Luminous efficiency (LE) is regularly mentioned as candela per ampere (cd/A) and measure the luminous intensity (in candela, cd), or luminance (L, in candela per meter square cd/m^2^) validated by a device at any current density (J). Quantum efficiency (QE) of a device is actually a measure of the photons formed from the injected hole and electron pairs in the LED. Among them, the photons emitted outside the LED are associated with the external quantum efficiency (EQE) and the photons generated inside the LED are responsible for internal quantum efficiency (IQE). Power efficiency (PE) is characterized as lumen per watt (lm/W) and defined as luminous flux output (in lumen) per input power of this device. The color quality of the generated white light is judged by its CIE coordinates, color rendering index (CRI) and color correlated temperature (CCT). CIE coordinates is a way to define any color in terms of (x,y) coordinates and is used to accurately represent every single color that a human eye can perceive. For e.g. the CIE coordinate of pure white light is (0.33, 0.33). CRI is used to measure the quality of a colored light as compared to that of the natural sunlight. It is usually measured in a scale of 0–100 and the CRI value of natural sunlight is assumed to be 100. CRI of a light source indicates how closely it can illuminate an object as compared to that of natural sunlight and replicate its true colors. It is therefore an important parameter to judge the quality of an artificial light source. In order to be used for indoor applications, a light source should possess high CRI value typically greater than 80. CCT of a light source is defined as the temperature of an ideal black body radiator radiating same light as that of the light source. It is usually used to define the color appearance of a WOLED. In general light with a CCT value of 2700 K or less, 4000 K and 5000 K or more is considered as warm light, neutral light and cool light respectively.

## Methods for generating white electroluminescence

To be utilized as a preferred white light source, the output spectral range of an OLED must span the complete visible range (400–800 nm) and their spectral distribution range must match with real sun light. The dominating approach to achieve this, in general, is to carefully combine the electroluminescent materials, typically two or three, emitting within the complementary range of visible spectrum. In evaporated OLEDs, this can be achieved by the fabrication of multilayer devices where each layer emits a specific color. However, in case of solution processed WOLEDs, white light is basically generated by single layer of a white-emitting copolymer where the emission of several colors takes place in a single layer. In this regard, several approaches such as doping green and red or orange emitting small molecules into a blue emitting polymeric host, polymer–polymer blending, electroplex, single white emitting polymer synthesized by incorporating green and red or orange moiety within the main/side alkyl chains of the blue light emitting conjugated polymer etc. has been the common strategy. Solution processed WOLEDs can also be fabricated using multilayer device structure, however, fabrication of multilayer polymer LEDs is more cumbersome as multilayer devices are difficult to process from solution and there is always a possibility that the solvent used in solution processing may destroy the layer beneath it. Fortunately, all these issues can be solved by proper selection of materials and solvents.

In case of TADF based OLEDs, tremendous effort has been devoted to generate white light by adopting TADF emitters, since they have higher triplet energy and high full width at half maximum (FWHM), enabling to transfer full energy either to counter green and red fluorescent emitter or by complementary yellow emitter. To suppress the carrier recombination in the device TADF emitters are being explored as assistant dopant and host, through FRET or exciplex formation mechanism. Remarkably, low cost solution processed TADF-WOLEDs fabricated by strategically employing single and multi-layer TADF emissive layer were found to demonstrate very low turn on voltages (V), high power efficiencies (PE (lm/W)), high current efficiencies (CE (cd/A)) and a maximum of 28% external quantum efficiencies (ηEQE) with Commission Internationale de L’Eclairage (CIE) co-ordinate near to standard (0.33, 0.33), extending its application towards a promising lighting technology for future generation WOLEDs.

## WOLEDs based on blending approach and exciplex formation

Blending small molecules or polymers emitting in the complementary region within 400 to 800 nm range is considered to be the simplest approach to fabricate white light emitting PLEDs. In this approach, a blue polymer material is usually used as a host material which is doped with green and orange/red light emitting small molecule or polymer to generate white light. Although this approach provides certain advantages such as easy control of the dopant concentration, easy synthesis process etc., it has drawbacks with respect to phase separation of the different dopants, complicated energy transfer as well as deep trapping processes during device operation leading to voltage dependent electroluminescence spectra.

**Fig. 1:**
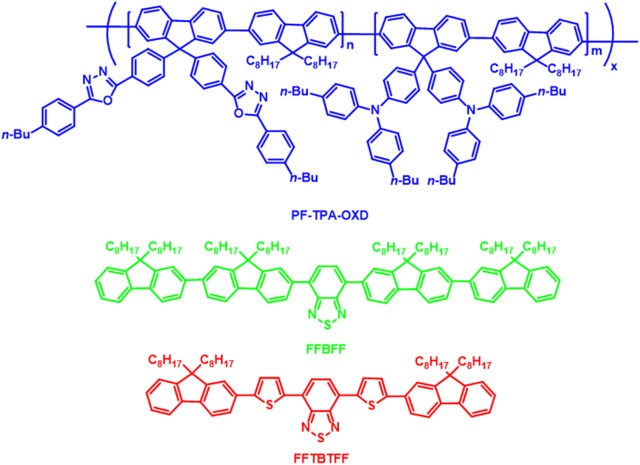
Molecular structures of the PF-TPA-OXD, FFBFF and FFTBTFF

A series of WOLEDs were fabricated by Kim et al. in 2004, by physical mixing of two fluorene derivatives as green (FFBFF) and red (FTBTF) emitting molecules into modified PFO copolymer (PFTPA-OXD). To obtain efficient charge balanced blue emitting PFO host the triphenyl amine moiety as hole and oxadiazole moiety as electron transporting materials were introduced into PFO main chain (Fig. 1). The ratios of the green and red emitting molecules were carefully controlled to achieve white emission. The WOLEDs in the architecture ITO/PEDOT:PSS/active layer/Ca/Ag were developed and these devices displayed highest brightness and EQE of 0.82% and 12,900 cd/m^2^, at 12 V [16]. CIE coordinates of these white light device was (0.36, 0.37) at 6 V to (0.34, 0.34) at 12 V.

**Fig. 2:**
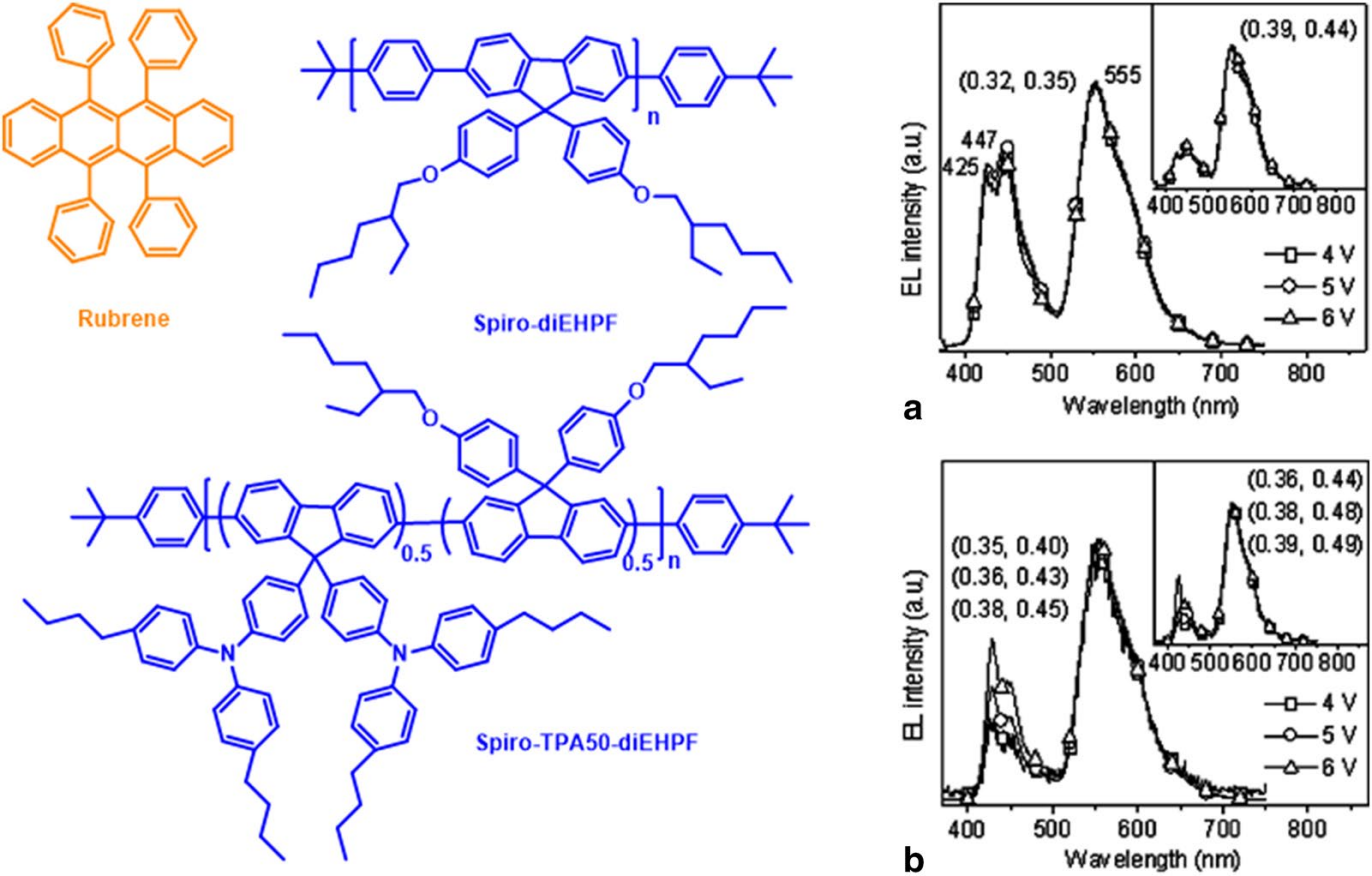
Chemical structures of rubrene and polymers. EL spectra of **a** rubrene-doped spirodiEHP (0.35 wt%) and **b** spiro-TPA50-diEHP devices. The insets in **a** and **b** EL spectra of rubrene-doped devices (0.5 wt%) (Reproduced with permission from Ref. [17] Copyright 2007, ©Applied Physics Letters)

WOLEDs were achieved by doping orange emitting rubrene into two different spiropolyfluorene derivatives such as spiro-diEHPF and triphenyl amine substituted spiro-polyfluorene (spiro-TPA50-diEHPF). White emitting and yellow emitting OLEDs were fabricated by 0.3% of rubrene into spiro-diEHPF and spiro-TPA50-diEHPF, respectively. These devices displayed a maximum brightness and luminous efficiencies of 56,000 cd/m^2^ and 9 cd/A for WLED and 72,000 cd/m^2^ and 14 cd/A for yellow LED. As shown in Fig. 2, the rubrene-doped spirodiEHPF based device displayed voltage independent EL spectra [17].

**Fig. 3:**
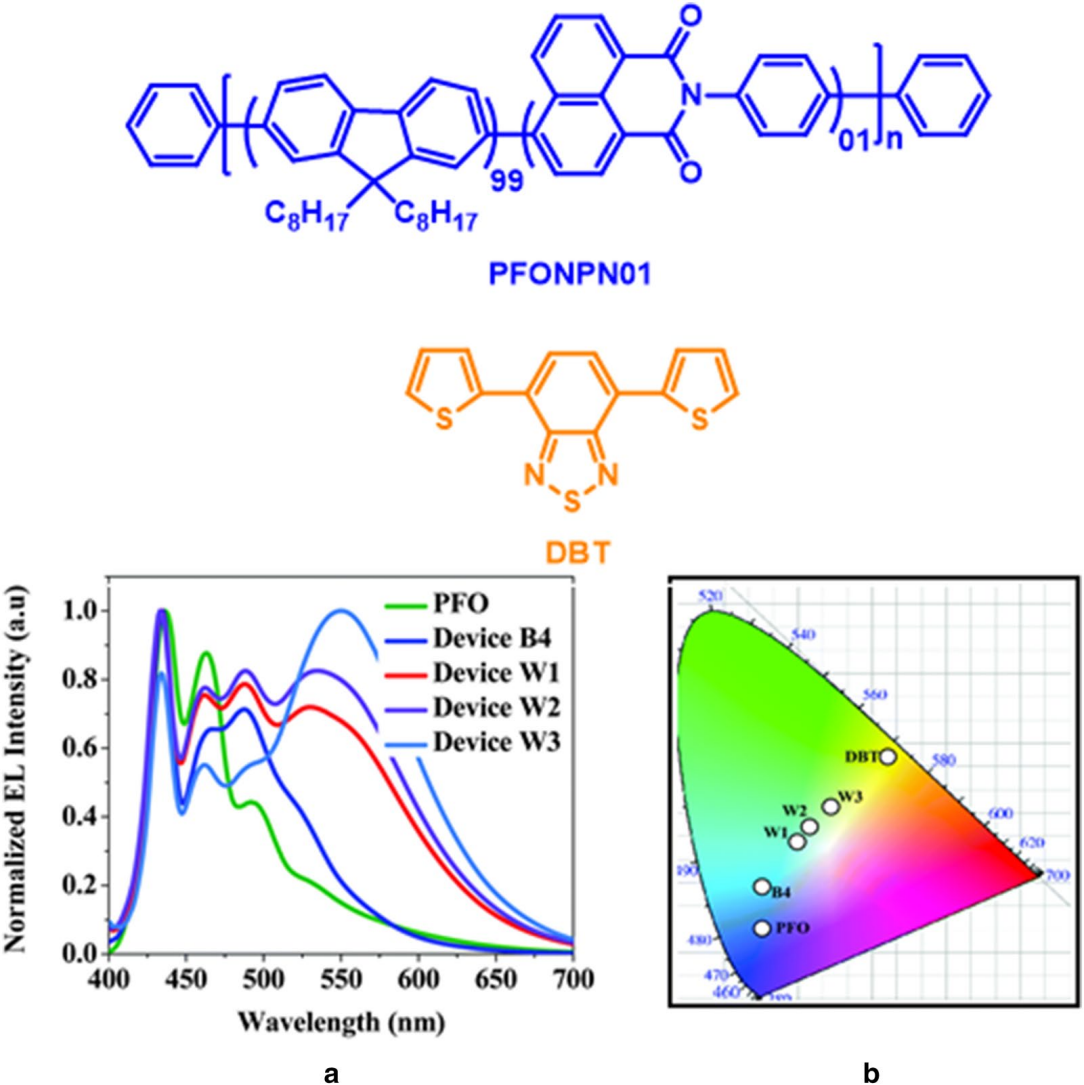
Molecular structures of PFONPN01 and DBT. **a** EL spectra and **b** CIE coordinate diagram of devices (B4-PFONPN01, W1, W2, and W3 with DBT ratio of 0.2%, 0.4% and 0.6%, respectively) (Reproduced with permission from Ref. [18] Copyright 2016, ©Physical Chemistry Chemical Physics)

Iyer and co-workers demonstrated efficient WOLEDs by doping of dithiophene benzothiadiazole (DBT) with an well charge balanced PFO derivative (PFONPN01), synthesized by introducing 1,8-naphthalimide derivative into PFO main chain (Fig. 3) [18]. The doping concentrations of the DBT was optimized and a very small amount of DBT (0.4%) concentration gave white light having CIE co-ordinates (0.31, 0.38). This white emission was obtained via incomplete FRET from the blue copolymer PFONPN01 to the orange emitting DBT guest. The fabricated WOLEDs had highest brightness value of 9565 cd/m^2^ and LE of 6.54 cd/A. EL spectra and CIE coordinate diagram of different doping concentrations of DBT (0.2% for W1, 0.4% for W2 and 0.6% for W3) is shown in Fig. 3.

**Fig. 4:**
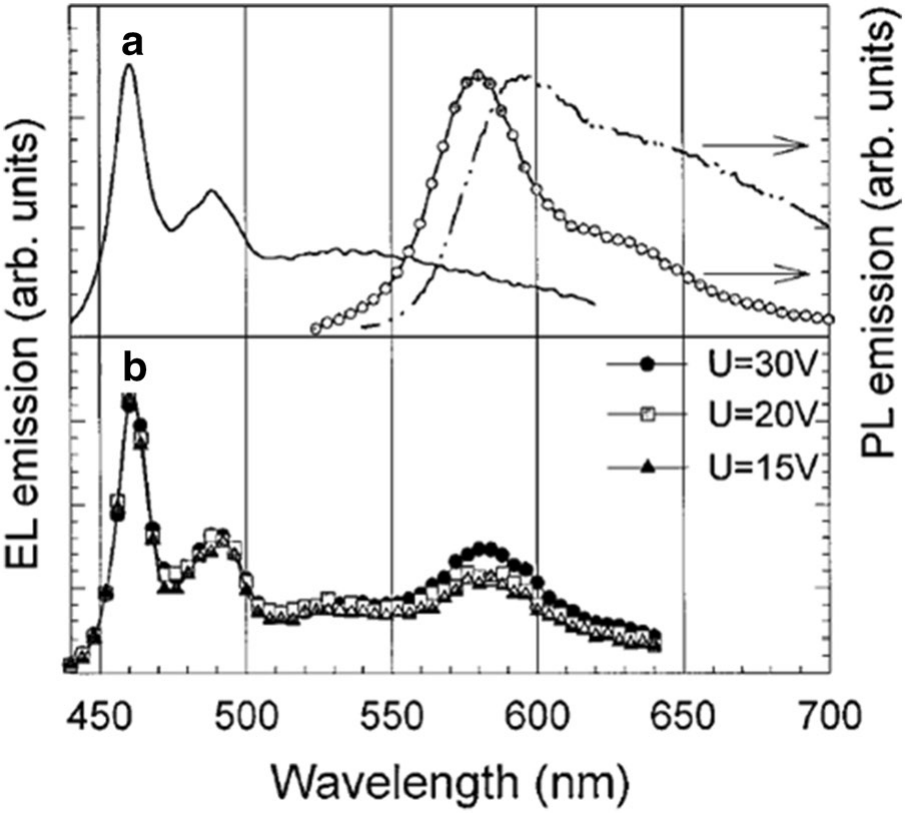
**a** EL spectra of *m*-LPPP (solid line) and the PL spectra of PPDB in solid state (dashed line) and solution form (circles). **b** EL spectra of the WPLED at different applied voltage (Reproduced with permission form Ref. [19] Copyright 1997 ©Applied Physics Letters)

The first WPLEDs using polymer blends was introduced in 1997 [19]. In this work poly-perylene-co-diethynylbenzene emitting in the red region was doped in very small quantity (0.05%) into a blue emitting polymer polyparaphenylene to form a homogeneous blend. Emission from both polymers were observed because of exciton energy transfer from the wide energy level blue emitting polymer to the narrow energy level red polymer which was responsible for emission of white light (Fig. 4).

By incorporating an insulating material PMMA into the polymer blend CIE coordinates of (0.31, 0.33) with an EQE of 1.2% was demonstrated. Although the addition of PMMA was found to hinder the energy transfer process from the blue to red polymer, better charge injection was observed due to the formation of dipoles within the PMMA matrix at the polymer-electrode interface. The addition of PMMA was also found to give rise to a voltage independent EL spectra of the WPLEDs.

Su et al. [20] in 2004, synthesized a blue emitting spiro-DPVF containing polyfluorene copolymer having voltage independent blue emission and realized white light by using MEH-PPV as the orange dopant. The efficient emission spectra overlap of the copolymer with the absorption spectra of the MEH-PPV along with the matching in the energy band position allowed Förster resonance energy transfer (FRET) to take place between the host and guest material along with the recombination as well as direct charge trapping along the MEH-PPV giving rise to white light. The WPLEDs were found to exhibit a maximum EQE value of 1.31% with brightness value of 956 cd/m^2^ and 8 V applied voltage whereas at 11 V applied voltage 3258 cd/m^2^ as highest brightness was observed. An almost stable EL spectra was also observed over the voltage range of 7–11 V.

**Fig. 5:**
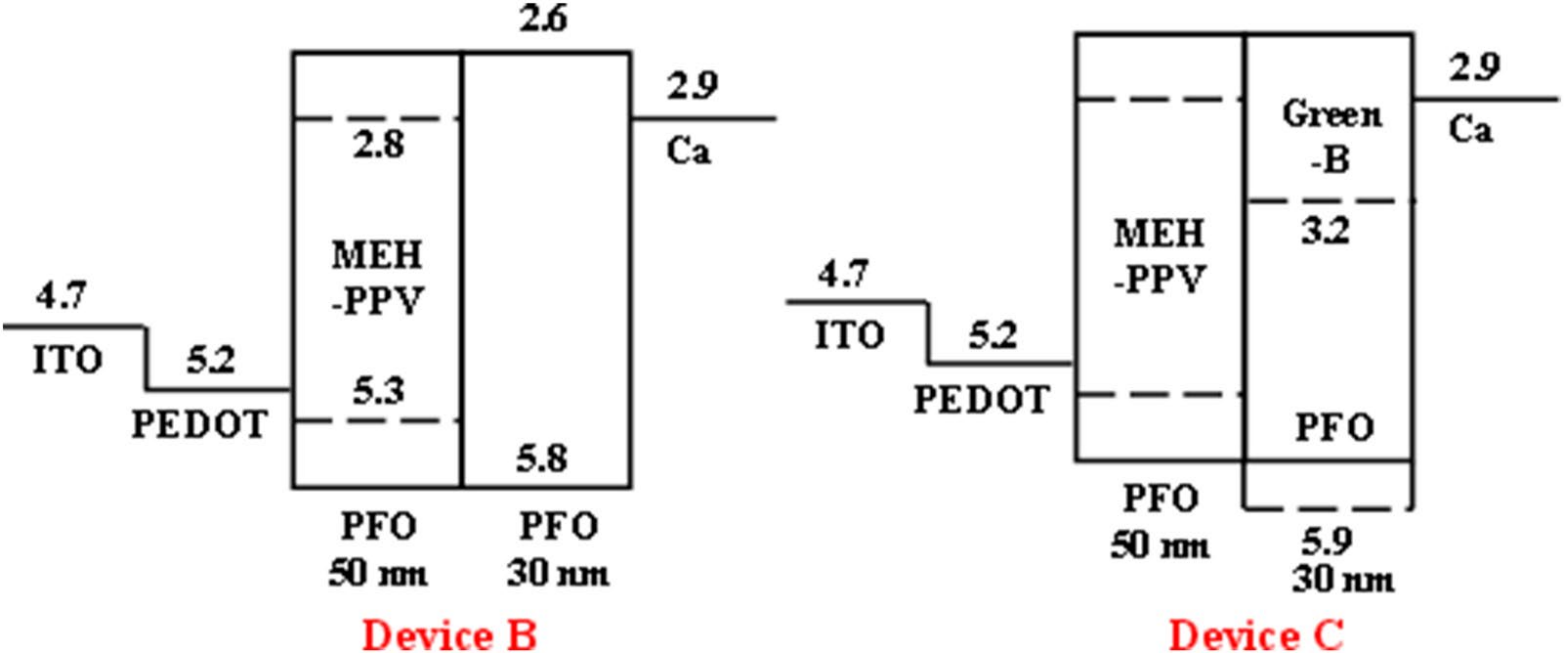
Energy band diagrams of the homojunction based WPLEDs (Reproduced with permission from Ref. [21] Copyright 2004 ©Applied Physics Letters)

Ho et al. [21] in 2004, utilized homo-junction between a blended polymer layer of PFO:MEH-PPV and pure PFO to realize white light. The energy band diagrams of the WPLEDs based on homojunction structure is compared and shown in Fig. 5. In their study, the blended layer was used to provide the orange emission and at the same to improve charge balance by utilizing it for hole transporting. They compared the properties of WPLEDs based on single blended polymer layer comprising of PFO and MEH-PPV with the homo-junction of PFO:MEH-PPV. In case of PFO:MEH-PPV blend, the EL spectra of the WPLEDs were MEH-PPV emission dominated due to efficient energy transfer. However, with increasing voltage the intensity of the PFO emission was enhanced with CIE coordinates (0.34, 0.35). The WPLEDs were found to give a highest brightness of 1700 cd/m^2^ at a luminous efficiency of 0.6 cd/A at 10 V. Compared to the single layer device, WPLEDs based on homo-junction was found to give lesser voltage dependent EL spectra along with a significant improvement in the brightness (3000 cd/m^2^) as well as efficiency (1.6 cd/A) of the devices. The weak voltage dependency of the EL spectra was due to the pure blue PFO layer emission irrespective of the applied voltage. However, the enhancement in the device parameters was due to the energy barrier created by the homo-junction that allows holes to accumulate in the MEH-PPV whereas electrons are distributed uniformly over the MEH-PPV and PFO. In order to be useful for full color display they further enhanced the green emission in the EL spectra by incorporation of a green emitting polymer Green-B to PFO.

Using the similar concept, Xu et al. [22] demonstrated WPLEDs with a CIE coordinate of (0.32, 0.32) over a large voltage range with considerable higher EQE and LE value of 3% and 4.4 cd/A respectively with brightness of 6300 cd/m^2^. They synthesized a red emitting copolymer comprising of PFO and 4,7-bis-3-hexyl-thien-2-yld-2,1,3-benzothiadiazole which was blended with PVK to form blend 1 and a phenyl-substituted PPV derivative (P-PPV) which was blended with PFO-poss to form blend 2.

**Fig. 6:**
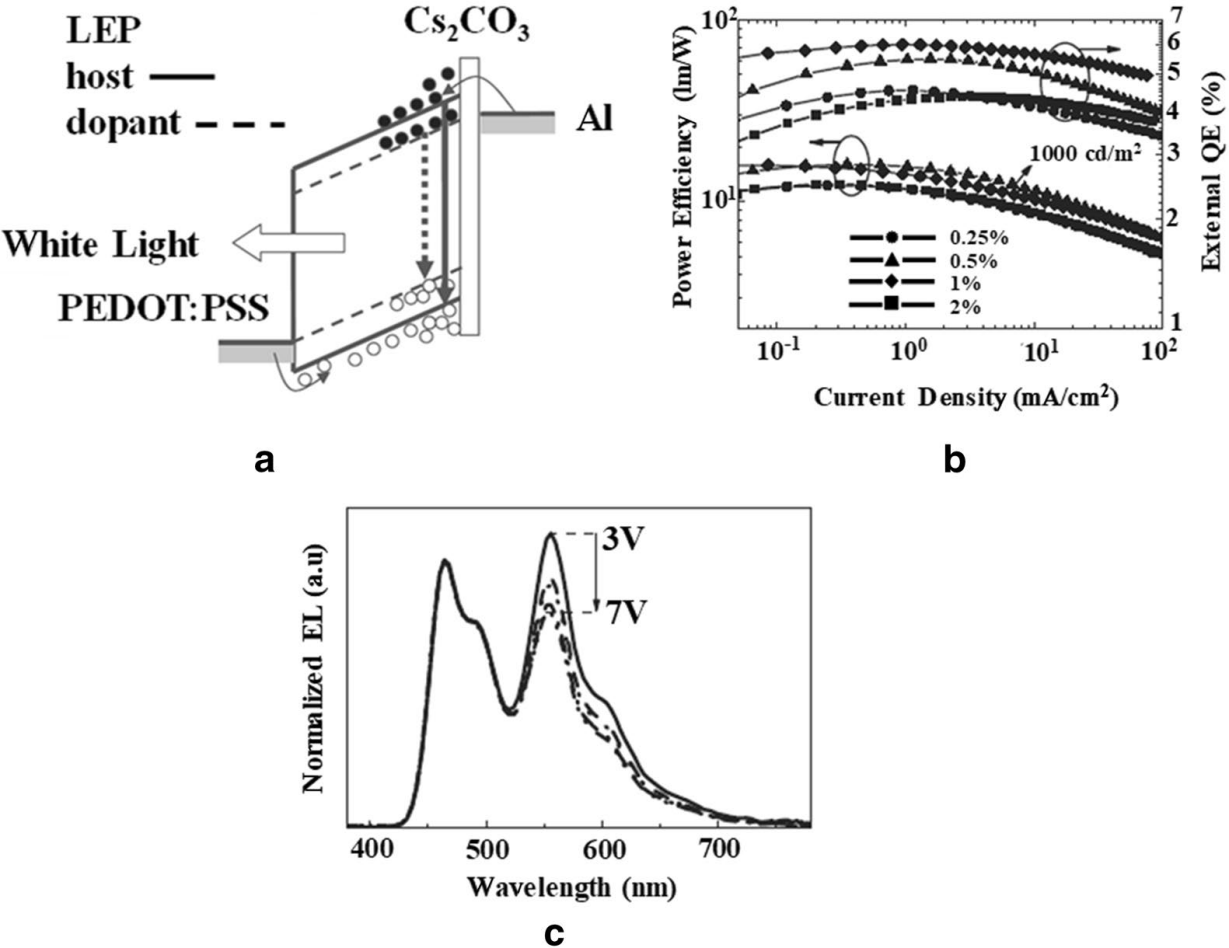
**a** Schematic energy band diagram of the WPLED. **b** External efficiency and power efficiency for the PLEDs with different doping ratio as functions of current density and **c** normalized EL spectra of MEH-PVV: PFO (0.25 wt%) PLED at different applied voltages (3 to 7 V) (Reproduced with permission from [23] Copyright 2006, ©WILEY–VCH Verlag GmbH &amp; Co. KGaA, Weinheim)

Huang et al. [23] in 2006, reported highly power efficient (16 lm/W) WPLEDs with polymer blends by introducing an interfacial Cs_2_CO_3_ layer between the polymer blend and cathode. MEH-PPV as an orange dopant was incorporated into PFO and white light was achieved via partial energy transfer and charge trapping mechanism. Figure 6 shows the device schematic, EL spectra and efficiency of the as fabricated WPLEDs. The addition of Cs_2_CO_3_ interfacial layer was found to significantly enhance the injection of the minority carriers from cathode resulting in a much-improved charge balance and hence the efficiency.

**Fig. 7:**
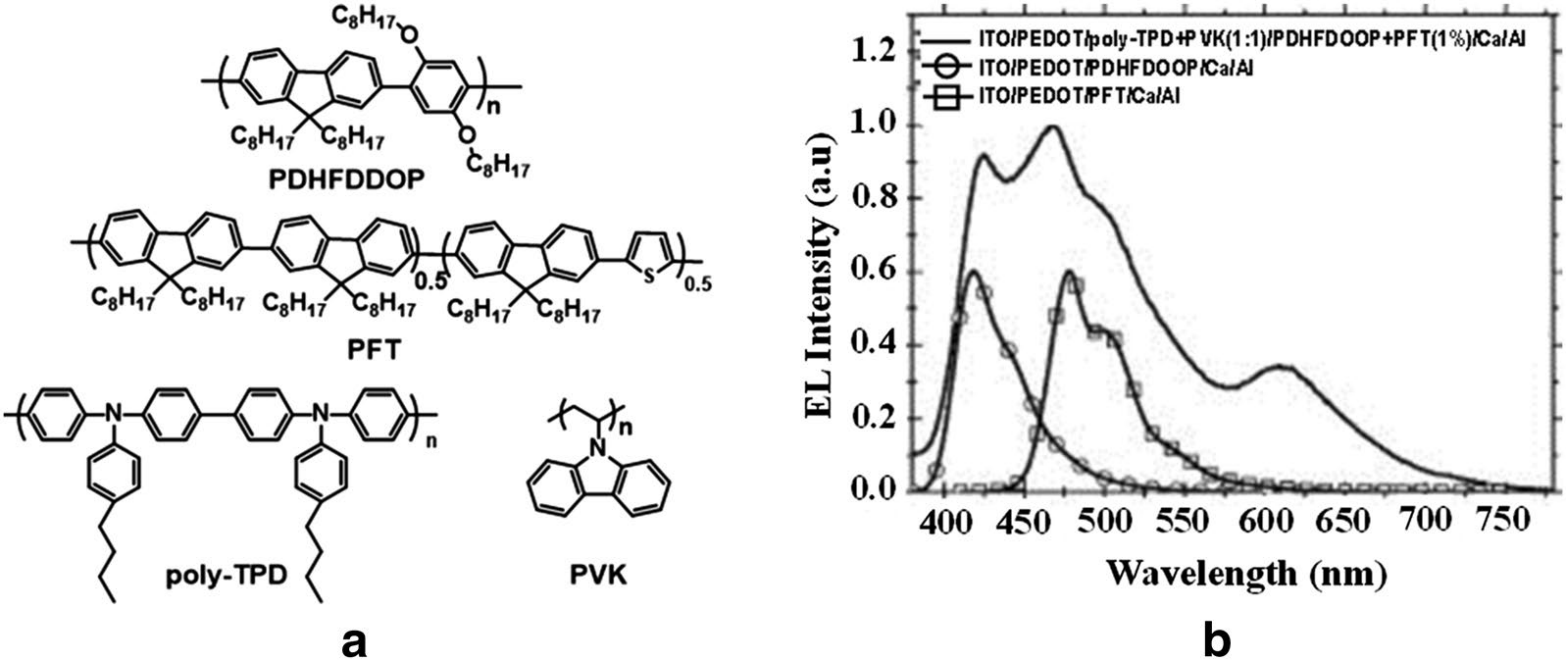
**a** Chemical structures of different polymers. **b** EL spectra of single and double layer PLEDs (Reproduced with permission from [24] Copyright 2006 ©Applied Physics Letters)

Sun et al. [24] in 2006, utilized the well-known fluorenone defects for the formation of exciplex between the hole transport layer with a blue and green blended luminescent polyfluorenes Poly(9,9-dihexylfluorene-altco-2,5-dioctyloxy-para-phenylene) (PDHFDOOP) and poly[6,6′-bi-(9,9′-dihexylfluorene)-co-(9′-dihexylfluorene-3-thiophene-5′-yl)] (PFT) to realize white light (Fig. 7). The as fabricated WPLEDs were found to show a highest brightness value of 4800 cd/m^2^ and a highest LE of 3 cd/A. Despite using only blue and green emitting polymers, the EL spectra of the devices exhibited low energy red emission which was assigned to the exciplex formation between poly-TPD, the hole transporting material and fluorenone defects produced in the PDHFDOOP due to the thermal annealing of EML while fabricating the device. These WPLEDs were found to emit white light with CIE coordinates close to (0.33, 0.33) over a wide voltage range of 9.5–17 V.

**Fig. 8:**
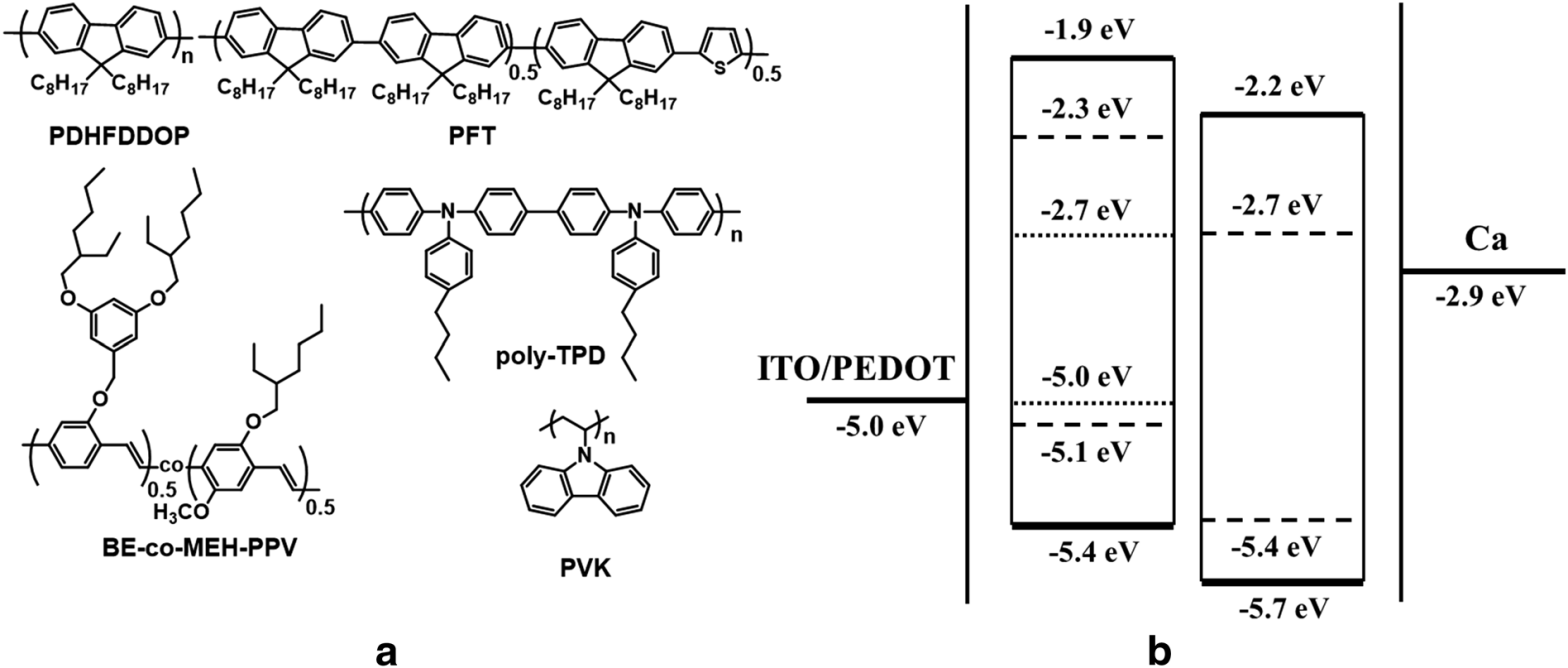
**a** Structures of polymers. **b** Energy level diagram of the WPLEDs [in BEL-PVK: solid line, poly-TPD: dashed line, and BE-co-MEH-PPV: dotted line; in TEL-PFO: solid line and PFT: dashed line] (Reproduced with permission from Ref. [25] Copyright © 2007, American Chemical Society)

Zhou et al. [25] utilized two-layer structure comprising of two blended emissive layer to avoid the most common color shifting problem of the WPLEDs based on blended systems and demonstrated voltage independent white light emission having (0.32, 0.34) as CIE coordinates over a voltage range 8–17 V. They used a mixed host of TPD and PVK for the bottom emissive layer and a red emitting BE-*co*-MEH-PPV as guest. PFO was used as the host material and top emitting layer along with green emitting PFT guest and by optimizing the weight ratios of the guest and host pure white light was achieved (Fig. 8). Poly-TPD was found to provide a greater resistance to the dissolving effect resulting in a smooth interface at the same time providing better hole injection due to its lower energy barrier with the PEDOT:PSS. Alternatively, due to the higher LUMO value of PVK as compared to that of PVK, it was able to confine the electrons within the bottom emissive layer. Therefore, utilization of a mixed host of poly-TPD and PVK was found to improve the efficiency of the device significantly from 1.8 cd/A (only PVK) to 4.4 cd/A. The selection of two polyfluorene derivatives PFO and PFT in the top emitting layer was also helpful to avoid the phase separation issue.

A series of highly fluorescent and thermally stable blue (B), green (G), and red (R) polymers based on 9,9-bis[4-(2 ethylhexyloxy)phenyl]fluorene (PPF) were synthesized [26] by incorporating a dibenzothiophene-*S*,*S* dioxide (SO) unit (PPF-SO polymer) together with either benzothiadiazole (BT) or 4,7-di(4-hexylthien-2-yl)benzothiadiazole (DHTBT). PLEDs were fabricated utilizing the emissive layers having the as synthesized PPF-SO25, PPF-SO15-BT1, PPF-SO15-DHTBT1. Highly efficient WPLEDs were realized on using different blend ratios of these polymers. Including an alcohol-soluble poly({9,9-bis[3′-(*N*,*N* dimethylamino)-propyl]-2,7-fluorene}-alt-2,7-(9,9-dioctylfluorene)) (PFN) EIL and a Blue/Green/Red blend ratio of 100/8/7 by weight, a maximum LE of 9.8 cd/A and PE of 8.9 lm/W was achieved. The WPLEDs with optimized device structures were found to demonstrate an excellent CRI of 90, and a CCT of 4700 K.

**Fig. 9:**
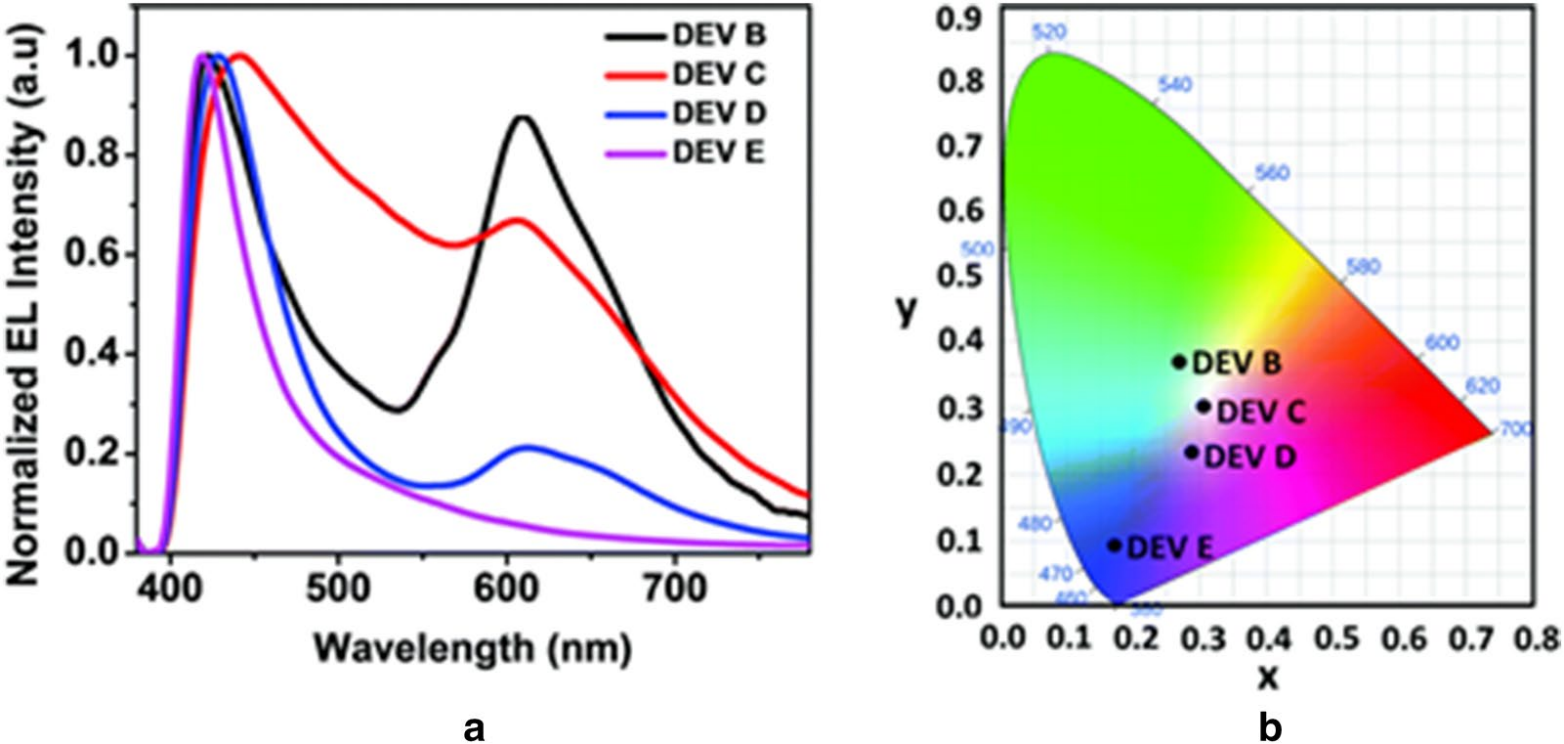
**a** The EL spectra and **b** the CIE co-ordinates diagram of the undoped PVK based PLEDs with or without different ETLs (DEV B-without ETL, DEV B-BCP, DEV B-Bphen and DEV B-TPBi) (Reproduced with permission from Ref. [27] Copyright 2016, ©Physical Chemistry Chemical Physics)

Das et al. realized white light emission through the electroplex formation. The EL spectra of the LEDs have been tuned by introducing the different electron transporting materials (ETLs). The PVK used as active layer and three different types of electron transporting materials were chosen, such as BCP, BPhen and TPBi. The electroplex peak obtained at 605 nm and the peak intensity was tuned with respect to the PVK emission. By introducing the different ETLs the device performance is drastically changed. The fabricated PLEDs had a device configuration of ITO/PVK/without ETL or BCP or BPhen or TPBi/LiF/Al. As shown in Fig. 9, the devices with ETLs BCP and/or BPhen gave close to white light emission with (0.30, 0.30) and (0.25, 0.23) as CIE values at 20 V [27].

Exciplex and electroplex are two different kind of excited state species that can be formed when recombination takes place between two adjacent materials. Usually exciplex or electroplex formation takes place between donor and acceptor moieties. The exciplex or electroplex generation is dependent on the material structure and the distance between the donor and acceptor and is smaller in case of exciplex as compared to that of electroplex. Electroplex observation can only be obtained in the EL spectra and not in the PL spectra whereas exciplex formation can be observed both in the EL as well as the PL spectra. Since these excited state species can be formed with any kind of materials such as fluorescent, or TADF emitters, hosts materials etc. WOLEDs based on exciplex and electroplex have been widely investigated by various research groups [28]. However, WOLEDs based on exciplex or electroplex usually suffers from the disadvantages of low color stability and poor lifetime.

## WOLEDs based on single layer copolymers

White emission can be generated from single polymer systems by chemically connecting/mixing of primary (Blue, Green and Red) or complementary (Blue and Orange) color emitting molecules, which are more beneficial than the previously existing methods. These molecular structures can provide easy solution processing for large area device fabrication and overcome the phase separation issue, which is more important to produce the stable pure white emission. These copolymers were mainly designed based on a dopant and host strategy, that required a small dopant quantity to be introduced into main chain or attached covalently to the host polymer side chain. The most challenging entity is that the mol% of dopant materials has to be appropriately maintained while introducing them into the host polymer backbone. It is noteworthy to mention that the very widely used polymer host is polyfluorene (PF), because of its high solid-state fluorescence quantum yield (0.55%) and good solution processing ability. The white emission can be realized through the partial FRET from wide band gap host molecule to low band gap dopant molecule. Numerous, WPLEDs have been reported so far, and among all, some of them are being discussed here based on their stable white emission and device performance.

**Fig. 10:**
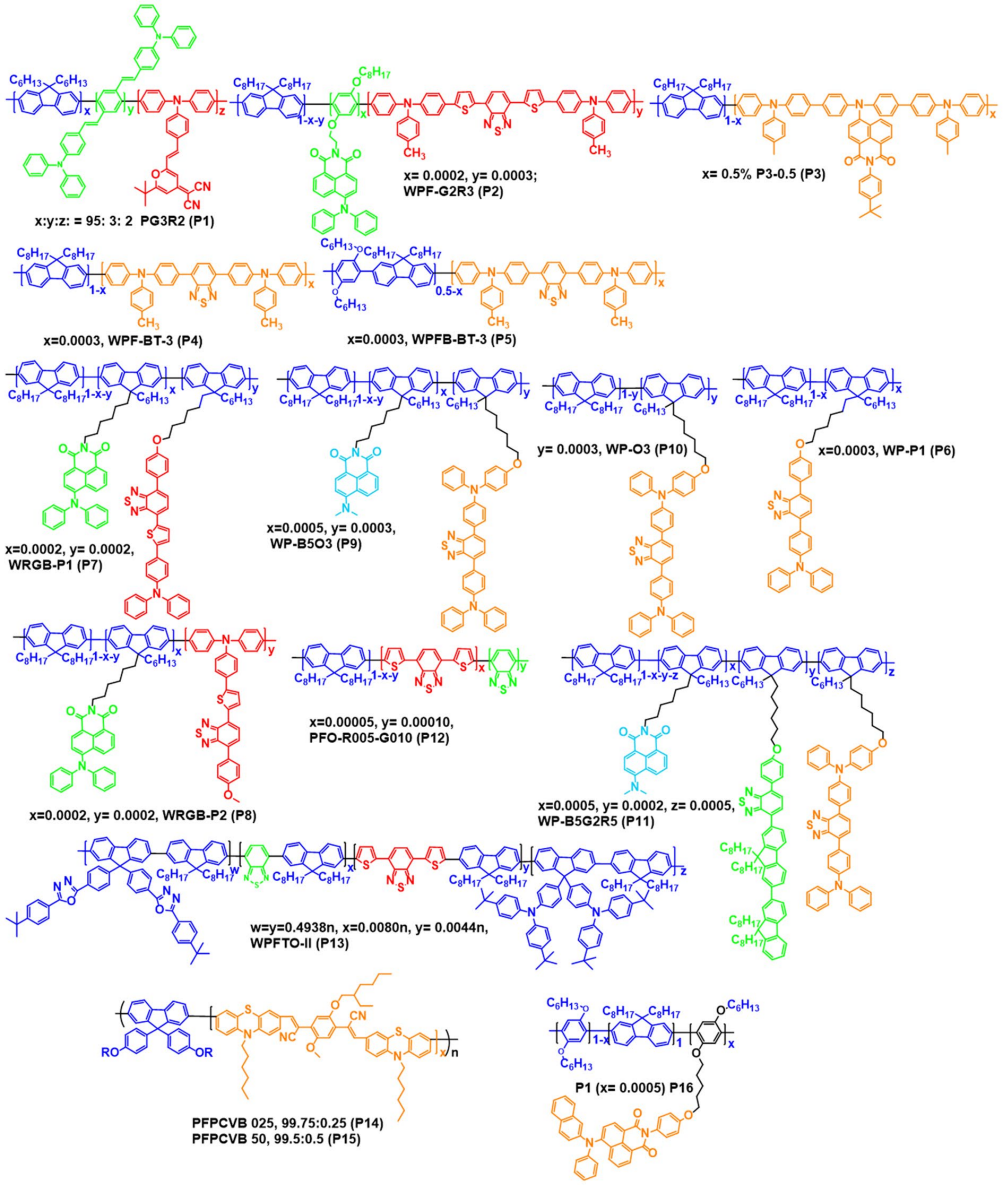
Chemical structures of the single layer copolymers

**Fig. 11:**
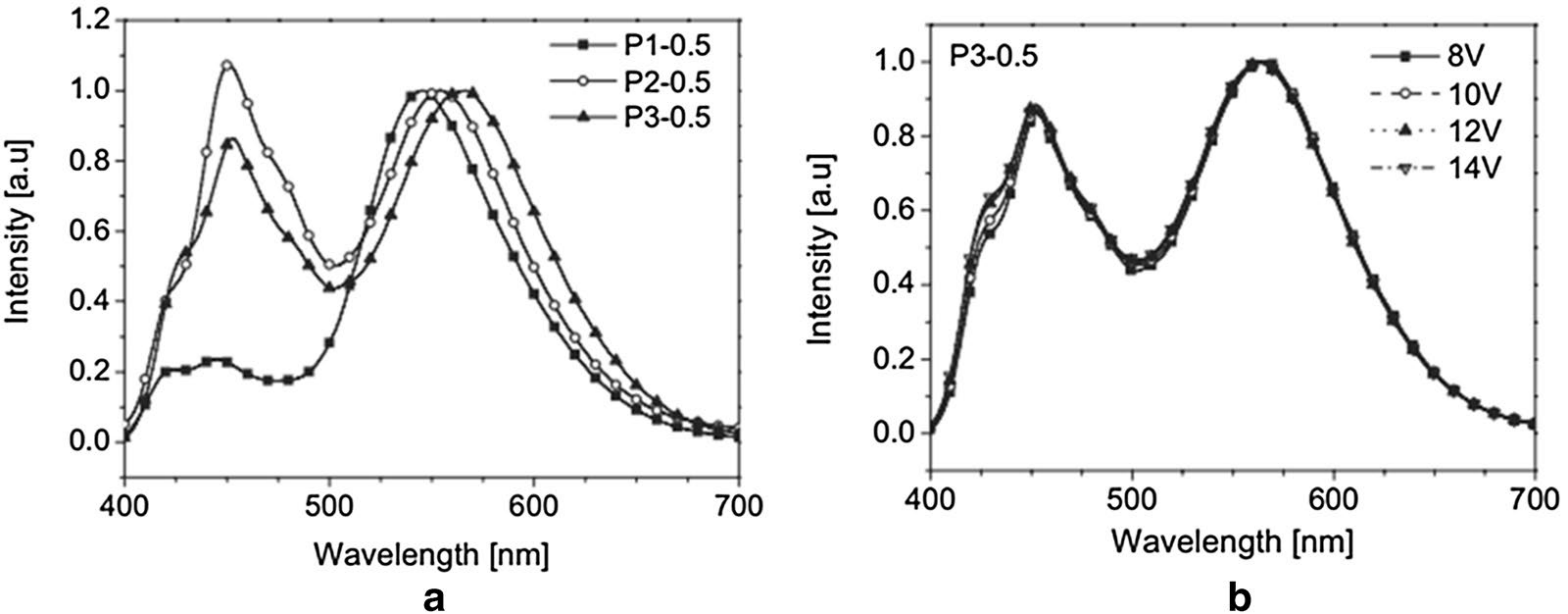
**a** EL spectra of the WPLEDs and **b** EL spectra of P3-0.5 at different driving voltages (Reproduced with permission from Ref. [31] Copyright 2005, ©WILEY–VCH Verlag GmbH &amp; Co. KGaA, Weinheim)

The first single polymer system has been designed and synthesized by Lee et al. in 2005, which consisted of primary color emitting materials (copolymer structures displayed in Fig. 10). The copolymer with feed ratio of 95(B):3(G):2(R) exhibited white emission and it displayed highest brightness of 820 cd/m^2^ and CIE coordinate values of (0.33, 0.35) at 11 V [29]. In the same year Liu et al. achieved white light emission from the single polymer system. The copolymers were synthesized by incorporating very small amounts of green dopant (0.0002 mol%) on the side chain and red dopant (0.0003 mol%) into the polyfluorene main chain. The WPLEDs were fabricated having configuration ITO/PEDOT:PSS/WPF-G2R3/Ca/Al (Table 1) [30]. In the pure single layer WPLEDs developed by Tu et al. in 2006, the copolymers were synthesized by inserting of 1,8-naphthalimide derivatives as orange dopants into the PFO backbone (P3) and the chemical structures are shown in Fig. 10. White emission was realized by adjusting of chemical feed ratios of the 1,8-naphthalimides in the PFO backbone. The single layer WPLEDs were fabricated having configuration ITO/PEDOT (50 nm)/polymer (80 nm)/Ca (10 nm)/Al (100 nm) (Table 1). Among the three polymers, triphenyl amine substituted copolymer exhibited very close to white light and additionally exhibiting highly stable EL spectra even on varying the driving voltages (Fig. 11) [31].

**Table 1:** EL performance of the single layer copolymers

Material	Device configuration	V_turn-on_ [V]	LE [cd/A]	EQE	Luminance [cd/m^2^]	CIE (x,y)	Refs.
P1	ITO/PEDOT:PSS/PG3R2/Ca/Al	6.0	0.10	–	820	0.33, 0.35	[29]
P2	ITO/PEDOT:PSS/WPF-G2R3/Ca/Al	5.8	1.59	–	3786	0.31, 0.34	[30]
P3	ITO/PEDOT/P3/Ca/Al	6.6	3.8	1.50	11,900	0.32, 0.36	[31]
P4	ITO/PEDOT:PSS/WPF-BT-3/Ca/Al	5.4	7.30	–	12,300	0.35, 0.32	[32]
P5	ITO/PEDOT:PSS/WPFB-BT-3/Ca/Al	6.6	2.53	–	3585	0.37, 0.34	
P6	ITO/PEDOT/WP-P1/Ca/Al)	3.5	10.66	–	21,240	0.30, 0.40	[33]
P7	ITO/PEDOT/WRGB-P1/Ca/Al	4.0	7.30	–	12,710	0.31, 0.32	[34]
P8	ITO/PEDOT/WRGB-P2/Ca/Al	4.0	3.80	–	12,870	0.30, 0.31	
P9	ITO/PEDOT/WP-B5O3/Ca/Al	3.5	12.8	–	18,480	0.31, 0.36	[35]
P10	ITO/PEDOT/WP-O3/Ca/Al	3.5	9.3	–	15,390	0.34, 0.34	
P11	ITO/PEDOT:PSS/WP -B5G2R5/Ca/Al	3.5	8.6	–	11,510	0.33, 0.36	[36]
P12	ITO/PEDOT/PVK/PFO-R005-G010/Ba/Al	8.88	2.97	–	472.77	0.32, 0.34	[37]
P13	ITO/PEDOT/WPFTO-II/TPBI/Mg:Ag	5.5	4.87	2.22	5000	0.37, 0.36	[38]
P14	ITO/PEDOT:PSS/PFPCVB 025/Balq/LiF/Al	–	0.34	0.41	860	0.30, 0.36	[39]
P15	ITO/PEDOT:PSS/PFPCVB 50/Balq/LiF/Al	–	0.97	0.90	1750	0.33, 0.39	
P16	ITO/PEDOT:PSS/P1/Ba/Al	–	22.62	–	–	0.30, 0.42	[40]
P17	ITO/PEDOT:PSS/FF-0.25/Al	4.1	5.17	3.10	9106	0.30, 0.31	[41]
P18	ITO/PEDOT:PSS/P(FCPA-0.5)/Al	–	4.6	–	5862	0.30, 0.33	[42]
P19	ITO/PEDOT:PSS/P(FCPA-1)/Al	–	4.5	–	6184	0.31, 0.32	
P20	ITO/PEDOT:PSS/FCP 2.5/Al	3.9	6.34	–	9332	0.33, 0.34	[43]
P21	ITO/PEDOT:PSS/FBPAN 0.5/Al	1.4	7.8	–	13,455	0.32, 0.31	[44]
P22	ITO/PEDOT:PSS/WDP-1/TPBi/LiF/Al	4.47	7.82	–	9753	0.31, 0.33	[45]
P23	ITO/PEDOT:PSS/WDT-1/TPBi/LiF/Al	4.14	4.57	–	7436	0.35, 0.34

**Fig. 12:**
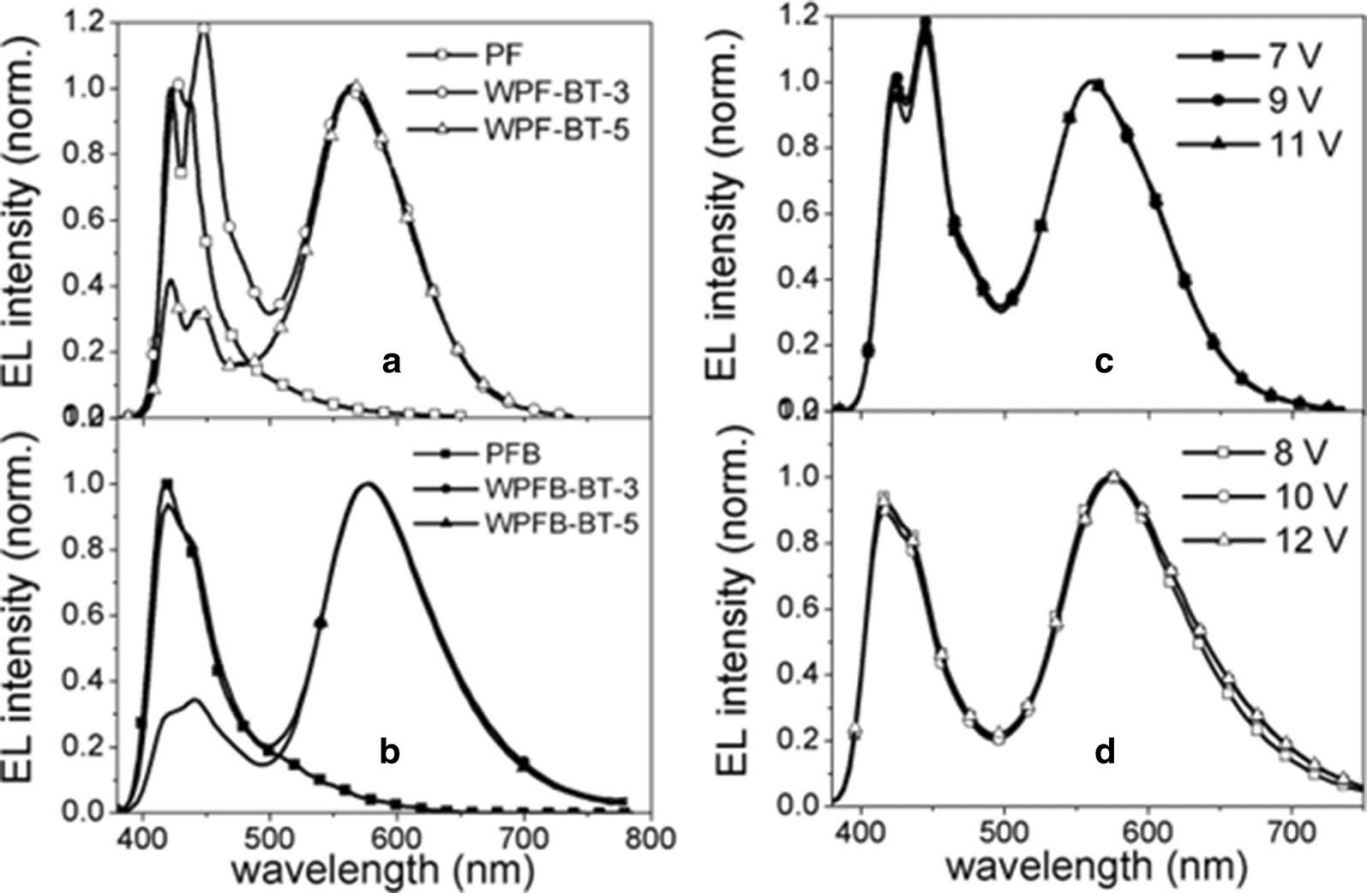
EL spectra of WPLEDs (**a**, **b**) and EL spectra of the WPLEDs, **c** WPFB-BT-3 and **d** WPF-BT-3 at different driving voltages (Reproduced with permission from Ref. [32] Copyright 2006, ©WILEY–VCH Verlag GmbH &amp; Co. KGaA, Weinheim)

In 2006 Liu et al. synthesized two sets of single layer white light emitting polymers, that consisted of PFO and poly(9,9-dioctyl-2,7-fluorene-alt-co-2,5-bis(hexyloxy)-1,4-phenylene) (PFB) as blue emitting host and orange emitting triphenyl amine substituted 2,1,3-benzothiadiazole (TPABT) as dopant material [32]. The chemical structure of the copolymers are shown in Fig. 10. The mol% of the TPABT was optimized to realize the white emission. The resulting copolymers were successfully fabricated into single layer WPLEDs (Fig. 12, Table 1).

Two sets of white single layer copolymers were synthesized by Wang and co-workers in 2007, in which the two copolymers exhibited the orange dopant covalently attached at side chain and remaining two in main chain of the PFO host (Fig. 10). The resulting copolymers were successfully utilized for WPLEDs fabrication and their device performances explored. The comparative studies revealed that the side chain type of copolymers showed better device performance than that of main chain type copolymers. As summarized in Table 1, among all copolymers, the side chain type (WP-P1) copolymer exhibited efficient device properties [33]. The same group also synthesized single layer white emitting copolymers by introducing different orange, green and red dopants into the PFO and modified PFO hosts (PFB5). The chemical structures of the side type and main chain type of copolymers are displayed in Fig. 10. The single layer WPLEDs were fabricated with resulting copolymers and the results compiled in Table 1. Based on their studies it can be concluded that the covalently attached side chain type of copolymers are more efficient than the main chain type copolymers [34–36]. In the same year Luo et al. proposed a unique approach to achieve white light emission, which consisted of a very small feed ratio of green and red emitting monomers introduced into blue emitting PFO polymer (Fig. 10). The feed ratios of the co-monomers were optimized to achieve white emission by partial energy transfer and the copolymer (PFO-R005-G010) with feed ratios of 0.00005 and 0.00010 for green and red monomers, respectively, showed the white emission. The WPLEDs were fabricated using the resulting copolymers and PFO-R005-G010 displayed better device performance and the results are summarized in Table 1 [37].

Chuang et al. developed efficient white luminescent copolymers by introducing narrow band gap green and red luminescent monomers into bipolar PFO back bone. The mol% of the narrow band gap monomers was optimized to realize the white emission and the WPLED with WPFTO-II copolymer was found to exhibit good device performance. The device properties are shown in Table 1 [38]. As displayed in Fig. 10, Park et al. [39] synthesized single layer white luminescent polymers (PFPCVBs) using palladium catalysed Suzuki reaction by inserting orange (BPCVB) luminescent monomer into the modified blue emitting PFO (BOPF) back bone. The WPLEDs had the device architecture ITO/PEDOT:PSS/active layer/Balq/LiF/Al. Among all copolymers the white emission can be obtained from PFPCVB 025 and PCPCVB 050 copolymers (Table 1) [39]. In 2010, Segura and co-workers developed novel white luminescent copolymers from poly(fluorene-*alt*-phenylene) and covalently attached 1,8-naphthalimide as blue and orange emitting species, respectively. The white emission from this single layer WPLEDs has been achieved by adjusting the 1,8-naphthalimide feed ratio in the blue emitting polymer back bone and the properties presented in Table 1 [40].

**Fig. 13:**
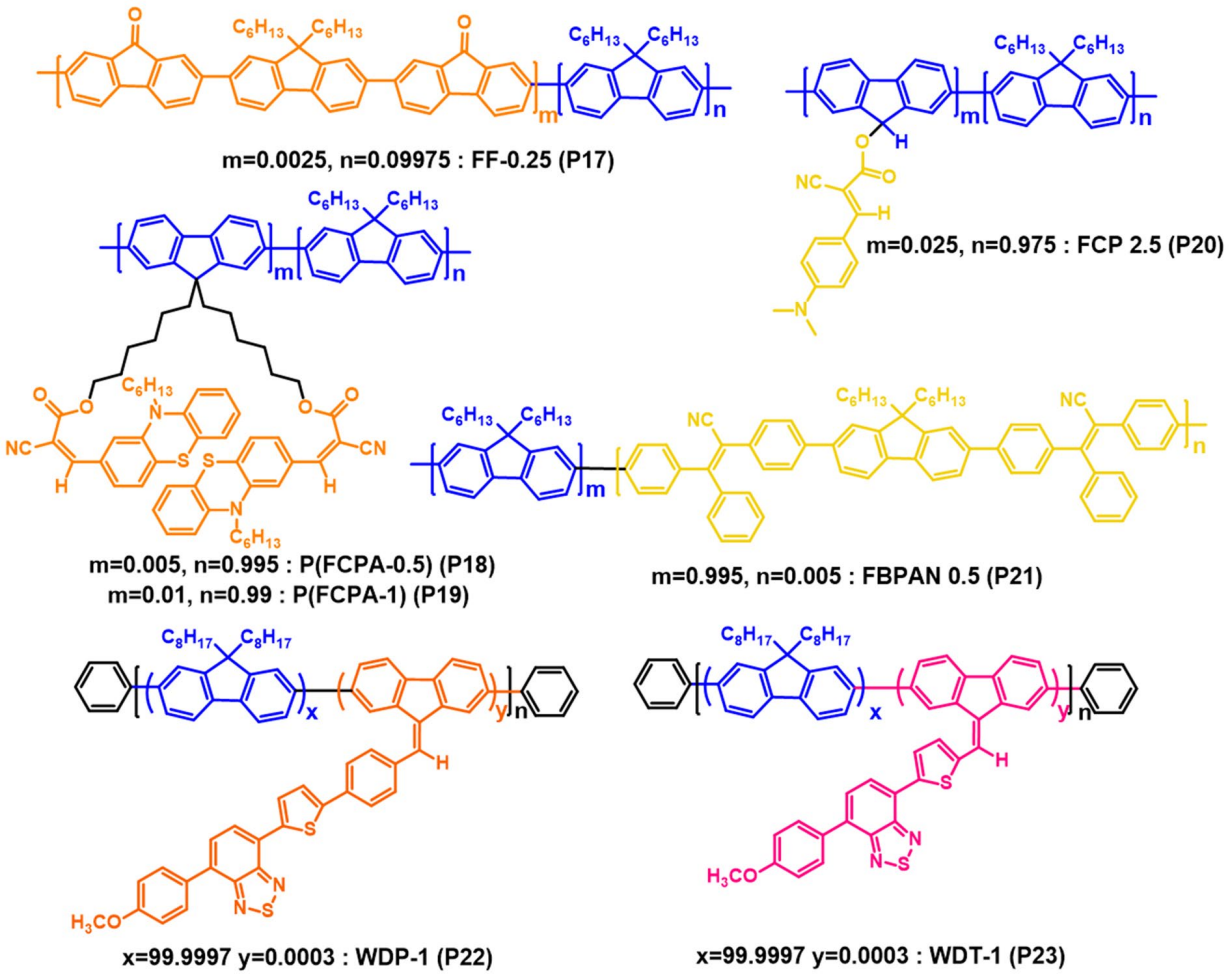
Chemical structures of AIEE active copolymers

Somanathan and co-workers described different types of single white emitting copolymers (Fig. 13), which composed of aggregation-induced emission enhancement (AIEE) luminogens as yellow or orange luminescent moieties and PFO blocks as the blue emitting host. To achieve the narrow bad gap AIEE active luminogens, donor and acceptor groups were combined together. The AIEE luminogens were introduced into PFO main chain as covalently attached pendant groups and as a main chain strategy. The efficient white luminescent copolymers were obtained via partial transfer of energy from the blue emitting wide band gap host to the yellow/orange emitting narrow band gap material by controlling the AIEE active luminogens feed ratios in the PFO back bone. The WPLEDs were constructed by solution processing the resulting copolymers with device architecture ITO/PEDOT:PSS/copolymer/Al. The device parameters of the WPLEDs are presented in Table 1 [41–44].

**Fig. 14:**
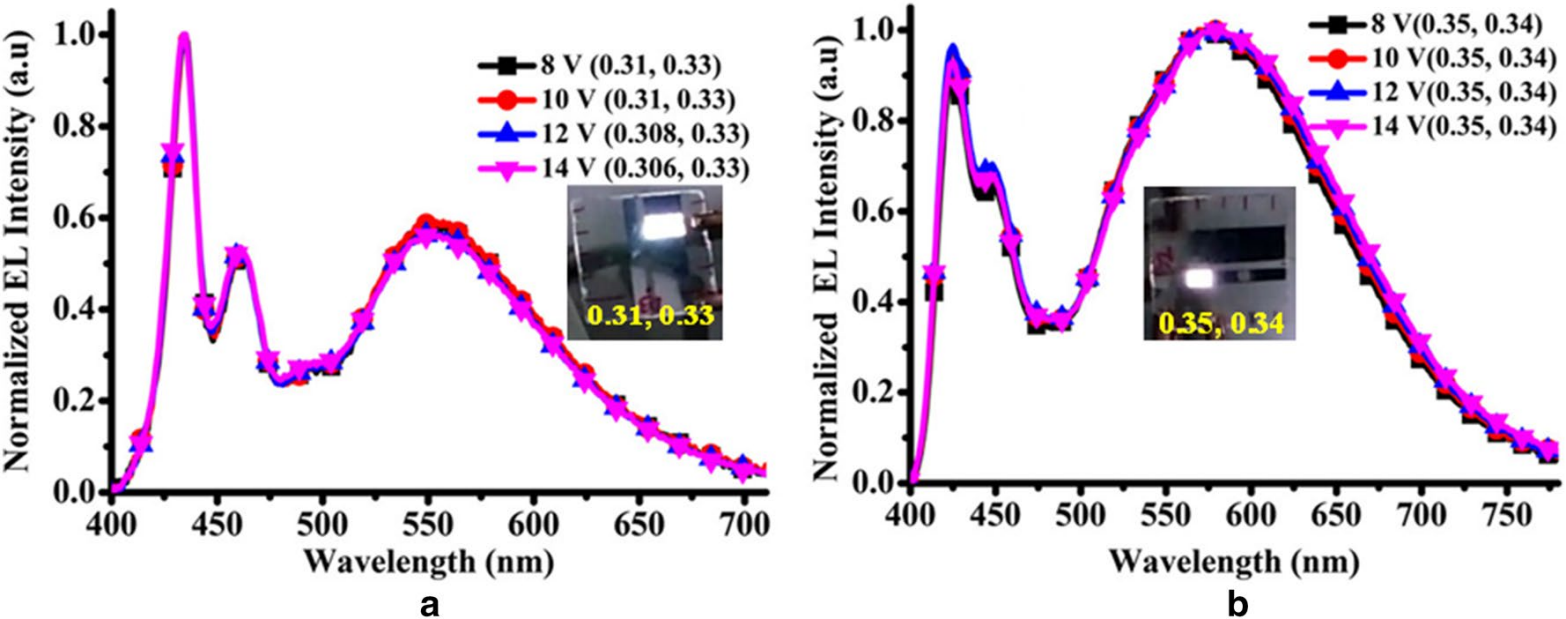
EL spectra of WDP-1 (**a**) and WDT-1 (**b**) copolymers based WPLEDs at different driving voltages (Reproduced with permission from Ref. [45] Copyright © 2017, American Chemical Society)

Recently, Iyer and co-workers designed and synthesized novel white electroluminescent copolymers by chemical incorporation of AIEE active mono-substituted dibenzofulevene (M-DBF) derivatives as orange/red emitting units into the PFO main chain (Fig. 14). The incomplete energy transfer has been easily obtained by controlling the mol% (0.0003% for the both WDP-1 and WDT-1 copolymers) of the AIEE orange/red luminogens in wide band gap blue emitting PFO back bone. Single layer WPLEDs having the architecture ITO/PEDOT:PSS/active layer/TPBi/LiF/Al have been fabricated and the data presented in Table 1. Importantly, when an enhanced voltage from 8 to 14 V was applied, exceedingly stable EL spectra of the copolymers WDP-1 and WDT-1 was obtained, which is very important for real world applications (Fig. 14) [45].

## WOLEDs based on TADF materials

### Förster resonance energy transfer (FRET) and exciplex TADF-WOLEDs

**Fig. 15:**
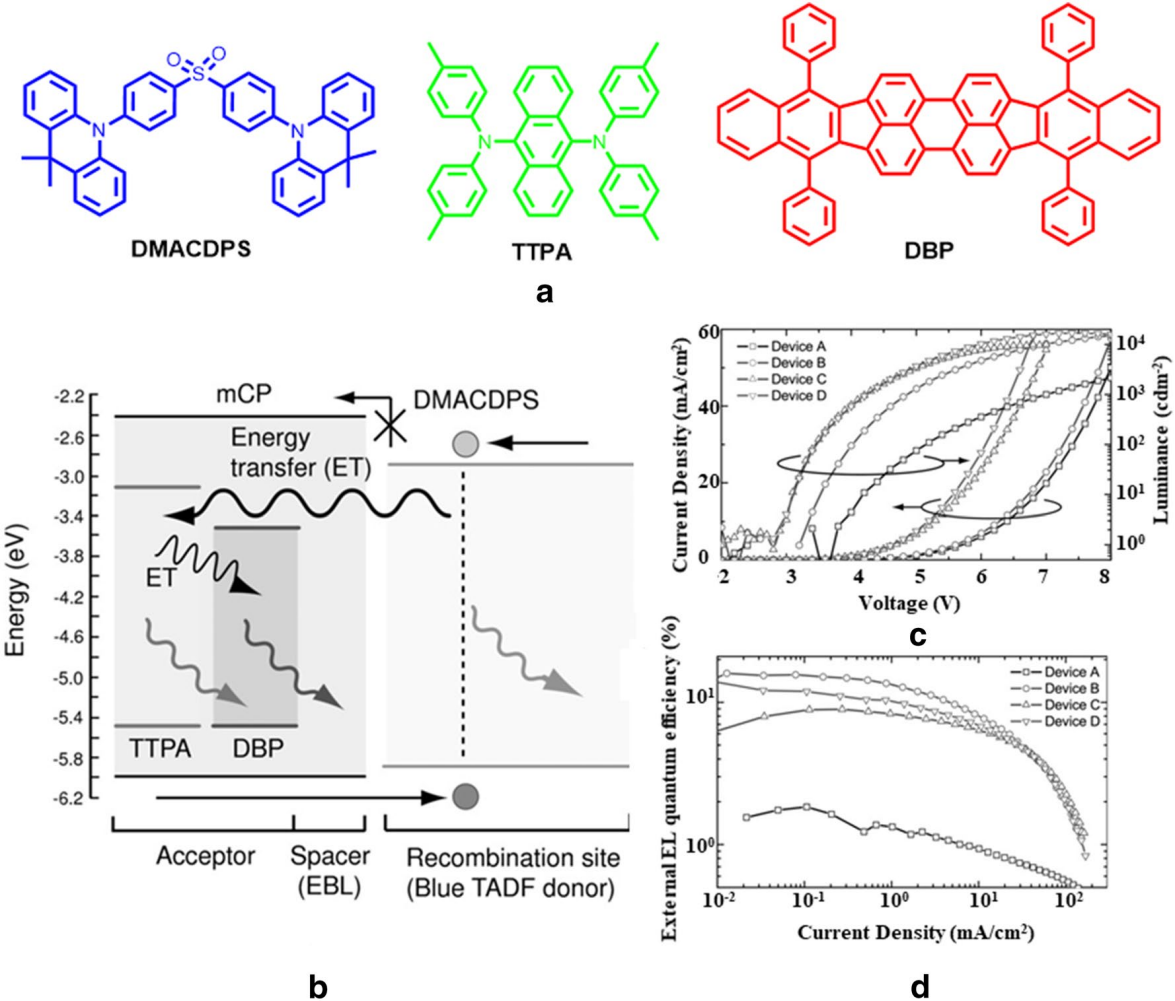
**a** Chemical structures of emissive materials, **b** FRET mechanism between DMACDPS and TTPA (green) and DBP (red) under electrical excitation [spacer layer mCP (2 nm)], **c** current density and luminance vs. voltage spectra and **d** external EL quantum efficiency vs. current density spectra of the OLEDs (Reproduced with permission from Ref. [46] Copyright 2015, ©WILEY–VCH Verlag GmbH &amp; Co. KGaA, Weinheim)

Adachi and co-workers pioneered a TADF-assisted fluorescence OLED (TAF-OLED) strategy by appropriate mixing of TADF with fluorescent molecules enabling promising operating stability and η_int_ of ~ 100% [46]. Especially, to achieve white light emission, a blue TADF molecule bis[4-(9,9-dimethyl-9,10-dihydroacridine)phenyl]sulfone (DMACDPS), acts as a common triplet harvester that has been employed with multiple traditional green and red 9,10-bis[*N*,*N*-di-(p-tolyl)-amino]anthracene (TTPA) and tetraphenyldibenzoperiflanthene (DBP) fluorophores respectively. Blue TADF emitter DMACDPS has higher triplet energy, and considered as exciton donor whereas fluorophores TTPA and DBP are exciton acceptors. Further, for realizing white light emission, a spatial separation was made by introducing 2 nm thick mCP layer to suppress major carrier recombination between TADF emitter and fluorophores. Interestingly, a long range FRET mechanism has been investigated where up-converted singlet exciton transfer energy to fluorescent emitter (shown in Fig. 15a, b).

Based on this above mentioned strategy, WOLED device has been fabricated (device architecture shown in Table 2). The device produces near white light having CIE coordinate values of (0.25, 0.31) at 1000 cd/m^2^ with 12.1% EQE and 22.0 lm/W luminous efficiency at 3.0 turn on voltage. Plots of (J)–(V)–(L) and ηEQE-EL curves for the devices are presented in Fig. 15c, d respectively.

**Table 2:** EL performances of different TADF materials

Material	Device structure	Turn on voltage (V)	CIE values	EQE (%)	Brightness (cd/m^2^)	LE (lm/W)	Refs.
DMACDPS TTPA DBP	ITO/α-NPD/DPEPO/DBP: TTPA: mCP/DMACDPS/TPBi/LiF/Al	3.0	(0.25, 0.31)	12.1	1000	12	[46]
NI-1-PhTPA PXZDSO2 DBP	W3: ITO/HATCN/TAPC/CBP: NI-1 PhTPA/CBP/CBP: PXZDSO2/CBP/CBP:NI-1 PhTPA/TmPyPB/LiF/Al	3.6	(0.38, 0.44)	15.8	5000	33.6	[47]
	W4: ITO/HATCN/TAPC/CBP: NI-1-PhTPA/CBP/CBP:PXZDSO2/CBP:PXZDSO2:DBP/CBP:PXZDSO2/BP/CBP: NI-1 PhTPA/TmPyPB/LiF/Al	3.4	(0.32, 0.40)	19.2	5000	47.5	
	W5: ITO/HATCN/TAPC/CBP: NI-1-PhTPA/CBP/CBP: PXZDSO2/CBP: PXZDSO2:DBP/CBP: PXZDSO2/CBP/CBP: NI-1-PhTPA/TmPyPB/LiF/Al	3.4	(0.33, 0.37)	17.3	5000	38.4	
Cz-4CzPN Cz-4CzTPN	ITO/PEDOT: PSS/Cz-4CzPN: Cz-4CzTPN/TPBi/Cs2CO3/Al	3.9	(0.34, 0.42)	17.3	11,000	30.4	[48]
AnbCz CDBP:PO-T2T	ITO/TAPC/TCTA/CDBP:PO-T2T:2CzPN:AnbCz/TmPyPB/LiF/Al	2.3	(0.34, 0.44)	19.0	1000	63.0	[49]
o,oʹ-NPh2 TXO-PhCz4	ITO/HAT-CN/TAPC/mCP/TXOPhCz4:o,oʹ-NPh2/TmPyPB/LiF/Al	3.7	(0.38, 0.40)	12.5	1000	27.1	[50]
SFI34pTz, DTPATXO	ITO/MoO3/NPB/mCP/DPEPO: SFI34pTz/SFI34pTz: DTPATXO/DPEPO: SFI34pTz/DPEPO/BPhen/LiF/Al	3.6	(0.32 ± 0.01, 0.42 ± 0.02)	22.9	500 to 3000	52.4	[51]
PTZ-TTR, PTZ-PhTTR	ITO/TAPC/TCTA/CBP:PTZ-TTR or PTZ-Ph-TTR/TmPyPb/LiF/Al	3.2, 3.45	(0.33, 0.33) (0.41, 0.47)	2.68, 16.34	300, 10,000	4.93, 41.75	[52]
DDCzTrz	ITO/MoO3/TAPC/DDCzTrz/TmPyPB/LiF/Al	3.0	(0.34, 0.35)	28.4	18 796	68.5	[53]

Li et al. reported a device structure by adjusting chromaticity tuneable concept exploiting TADF material PXZDSO2 in the device to achieve pure white OLEDs [47]. A white light emission has been realized by utilizing conventional deep-blue-fluorescence emitter NI-1-PhTPA, which comprises a blue emission from singlet excitons, while the triplet excitons give rise to transfer the energy into a longer wavelength PXZDSO2 TADF emitter. Furthermore, to accumulate the singlet excitons of PXZDSO2, a conventionally used deep-red-fluorescence emitter DBP was appropriately incorporated in the device to realize full visible region spectra and high CRI value.

**Fig. 16:**
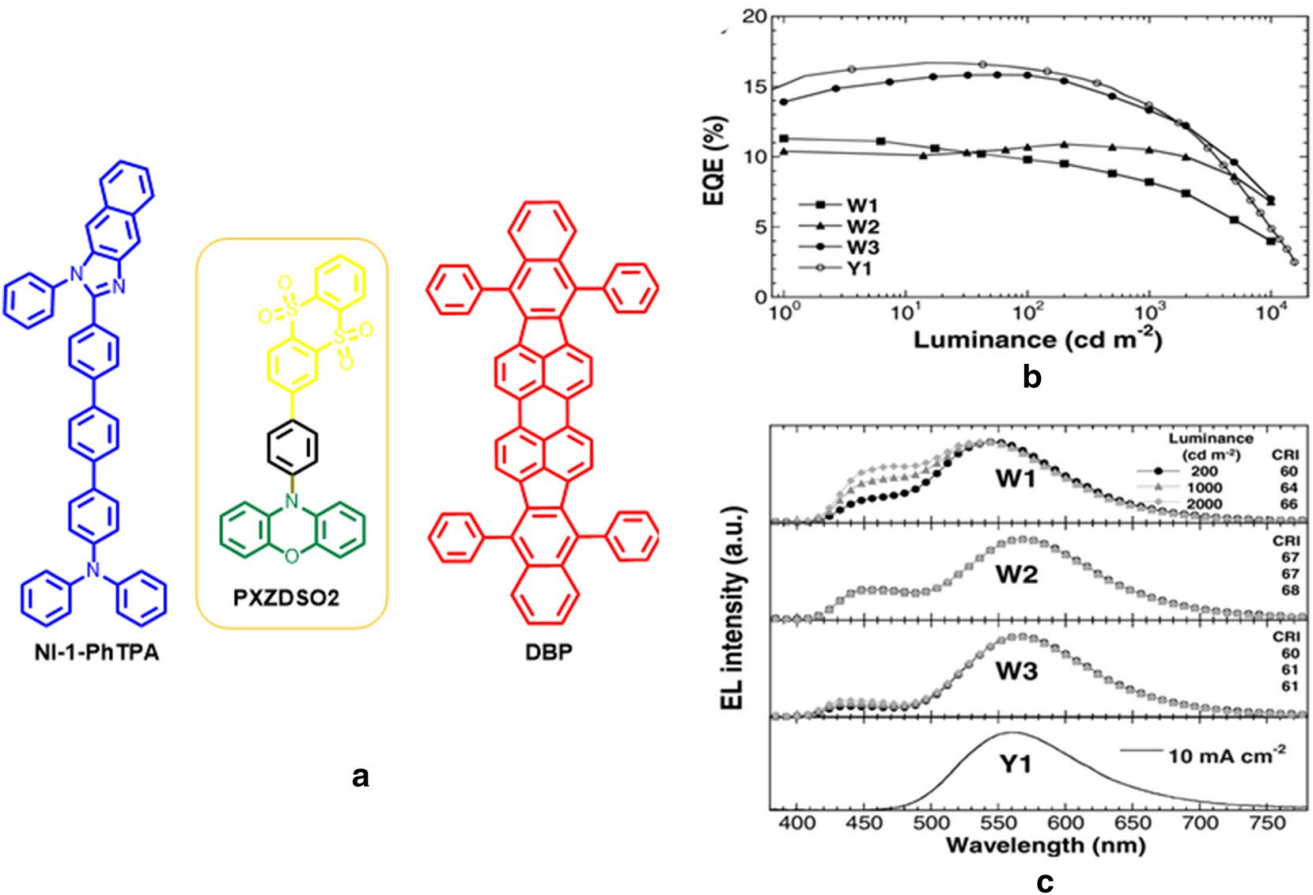
**a** Chemical structure of NI-1-PhTPA, PXZDSO2 and DBP, **b** EQE vs. luminance spectra and **c** EL spectra of the OLEDs W1, W2, W3, and Y1 (Reproduced with permission from Ref. [47] Copyright 2016, ©WILEY–VCH Verlag GmbH &amp; Co. KGaA, Weinheim)

Moreover, a series of white light emitting devices were developed by manipulating the excitons of singlet and triplet states using a sole yellow TADF emitter. Among all these devices W3 and W4 exhibited pure white light by employing two color pure organic emitter, devices results EQE of 15.8% and 19.2% with CIE coordinates (0.382, 0.448) and (0.322, 0.408) respectively, where two-color pure organic molecules inserted. Three color WOLEDs also made by adding DBP red emitter and a 15.6% EQE and 95 CRI value with CIE coordinate (0.332, 0.371) accomplished by a candle-style warm light OLED (Fig. 16).

Ban et al. explored an effective method for the first time by embedding a classical small TADF molecular structure for low cost solution processed WOLEDs [48]. To make a feasible solution processed device, two yellow TADF materials, Cz-4CzPN and Cz-4CzTPN were carefully developed. Without altering the TADF property an unconjugated alkyl chain was encapsulated. The molecular modulation and encapsulation effects have been investigated by fabricating solution-processed WOLEDs with a simple device architecture ITO/PEDOT:PSS (40 nm)/blue host: x% yellow dopant (60 nm)/TPBi (40 nm)/Cs_2_CO_3_ (2 nm)/Al (100 nm). Previously developed blue TADF Cz-3CzCN emitter was utilized as a host which generates instant blue fluorescence from partial singlet exciton decay and rest are transferred to the singlet state of yellow Cz-4CzPN and Cz-4CzTPN dopants, to produce yellow emission. A long range Förster energy transfer (FET) into yellow dopant or weak delayed fluorescence takes place from thermally up-converted 75% triple excitons through RISC process of the TADF blue host.

**Fig. 17:**
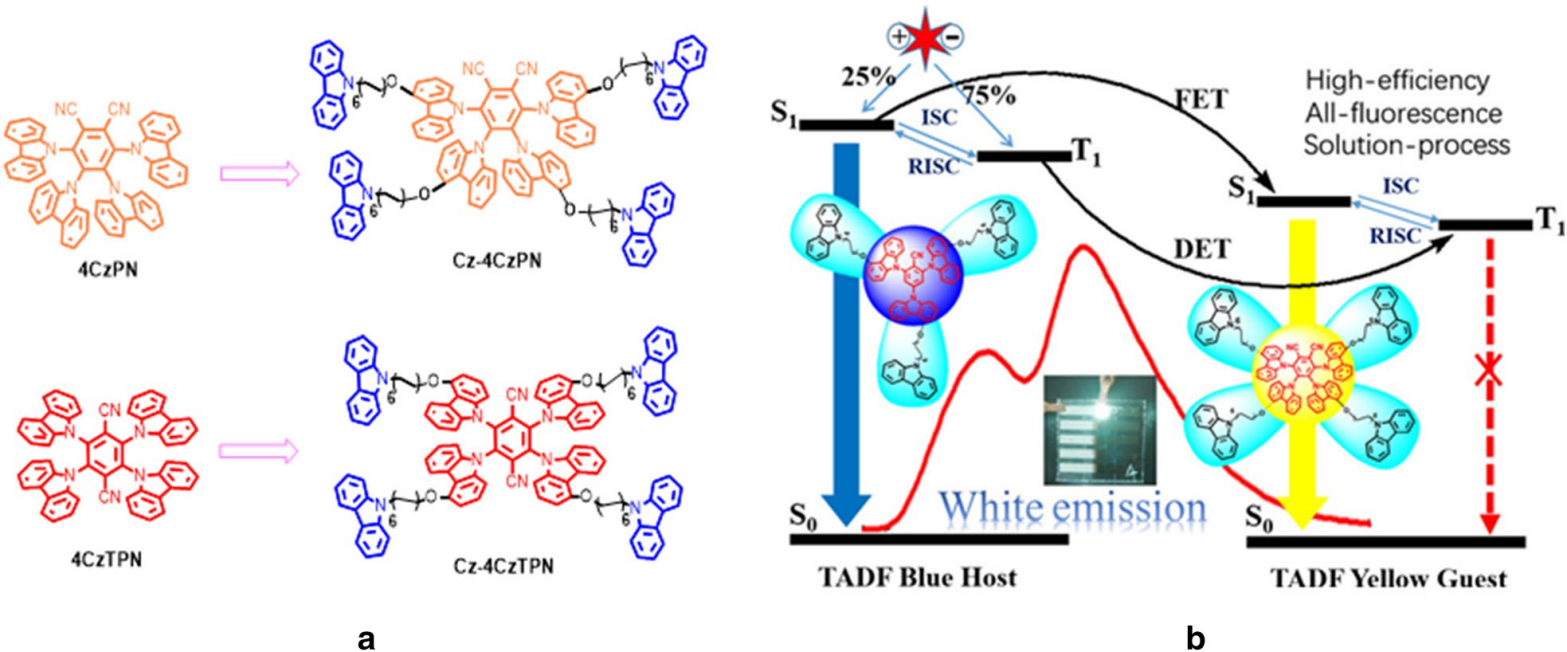
**a** Molecular structures of the emitters, **b** energy transfer mechanism and molecular stacking depiction (Reproduced with permission from Ref. [48] Copyright © 2018, American Chemical Society)

Excitingly, the losses of partly derived triplet excitons of blue host, through Dexter energy transfer (DET) or ISC process are also up-converted by yellow TADF emitter to achieve radiative decay (Fig. 17). Thus, the blue TADF host and the yellow emitting TADF material enables harvesting all the excitons to generate white light. Moreover, an all- fluorescence single-EML WOLED has been fabricated for the Cz-4CzPN with 0.6% optimized dopant concentration, and device achieved white light emission at (0.34, 0.42) CIE value, 41.6 cd/A current efficiency (CE), 30.4 lm/W power efficiency (PE), and 17.3% external quantum efficiency (EQE) respectively (Table 2).

**Fig. 18:**
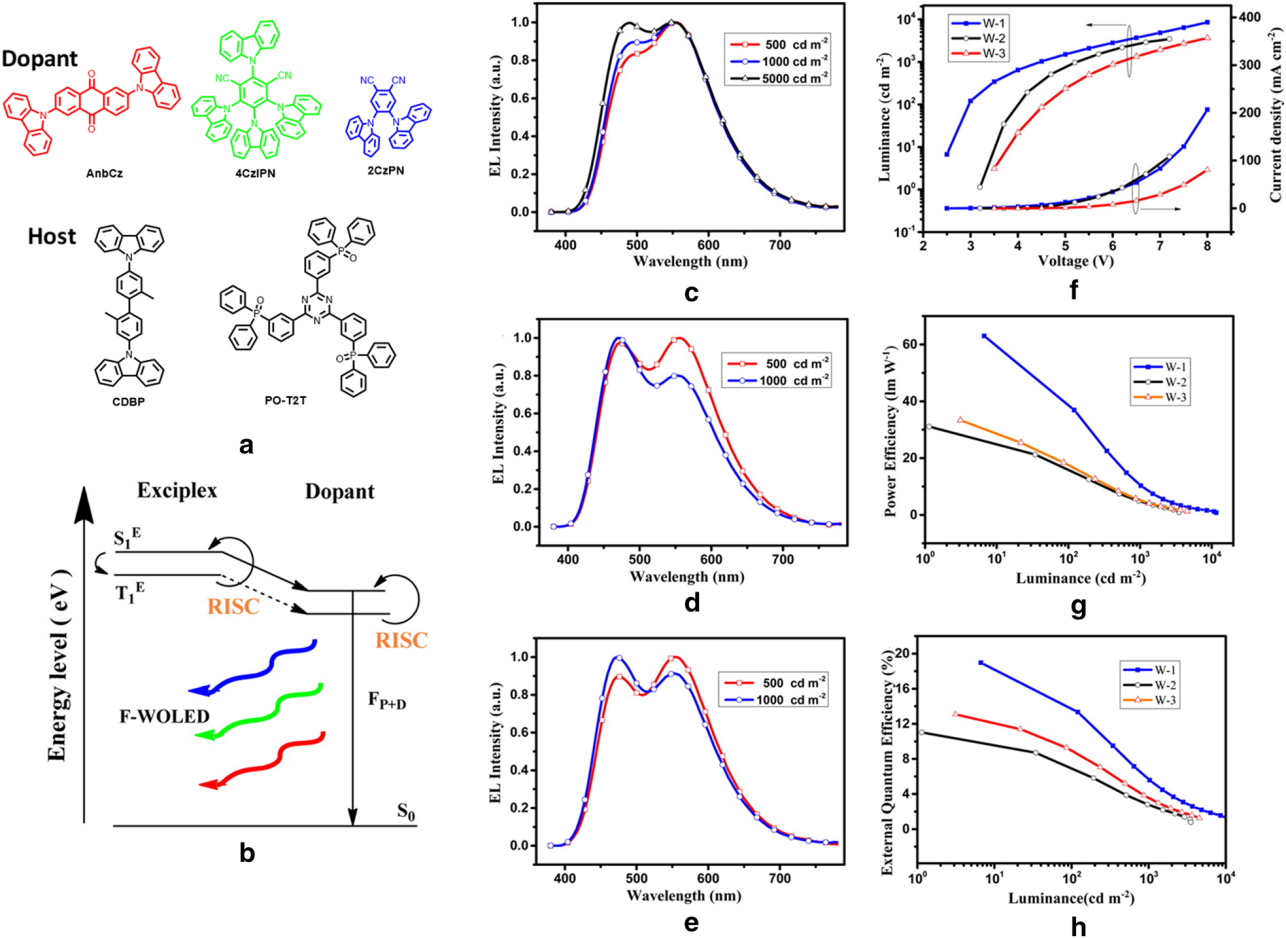
**a** Chemical structures of emitter dopants and hosts. **b** Energy transfer mechanism of the TADF emitters with exciplex host (solid arrow indicates FRET and broken line arrow indicates DET). EL spectra of the devices at varying luminance **c** devices W-1, **d** W-2, and **e** W-3, **f** current density (J–V) vs. luminance characteristics, **g** PE-luminance and **h** EQE vs. luminance plots of the three fabricated F-WOLEDs

Recently, Liu et al. established a TADF exciplex concept, and universal multi-colored host emitters, to achieve 100% energy transfer through either FET or Dexter energy transfer (DET) mechanism and a maximum up-converted triplet excitons from exciplex host to TADF emitter dopant [49]. Moreover, the carrier transporting property of TADF exciplex, released carrier injection barrier to EML, enabling an effective energy donor to wide range of dopants. TADF exciplex CDBP (4,4′-bis(9-carbazolyl)-2,2′-dimethylbiphenyl):PO-T2T ((1,3,5-triazine-2,4,6-triyl)tris-(benzene-3,1-diyl)tris (diphenylphosphine oxide)) were selected as host for blue-2CzPN, green-4CzIPN and orange-AnbCz TADF emitters (Fig. 18a–h).

A F-WOLED device was constructed having the structure: ITO/TAPC (40 nm)/TCTA (10 nm)/CDBP:PO-T2T: 7.5 wt% 2CzPN: 0.6 wt% AnbCz (30 nm)/TmPyPB (45 nm)/LiF (1 nm)/Al (100 nm) without additional charge transporting materials. A simplified electroluminescent device exhibited highly efficient white emission, with rather conventional host having maximum EQE, PE, current efficiency (CE) of 19.0%, 63.0 lm/W, 50.1 cd/A at much lower turn-on voltages reported among F-WOLEDs.

### Multi-layer TADF-WOLEDs

**Fig. 19:**
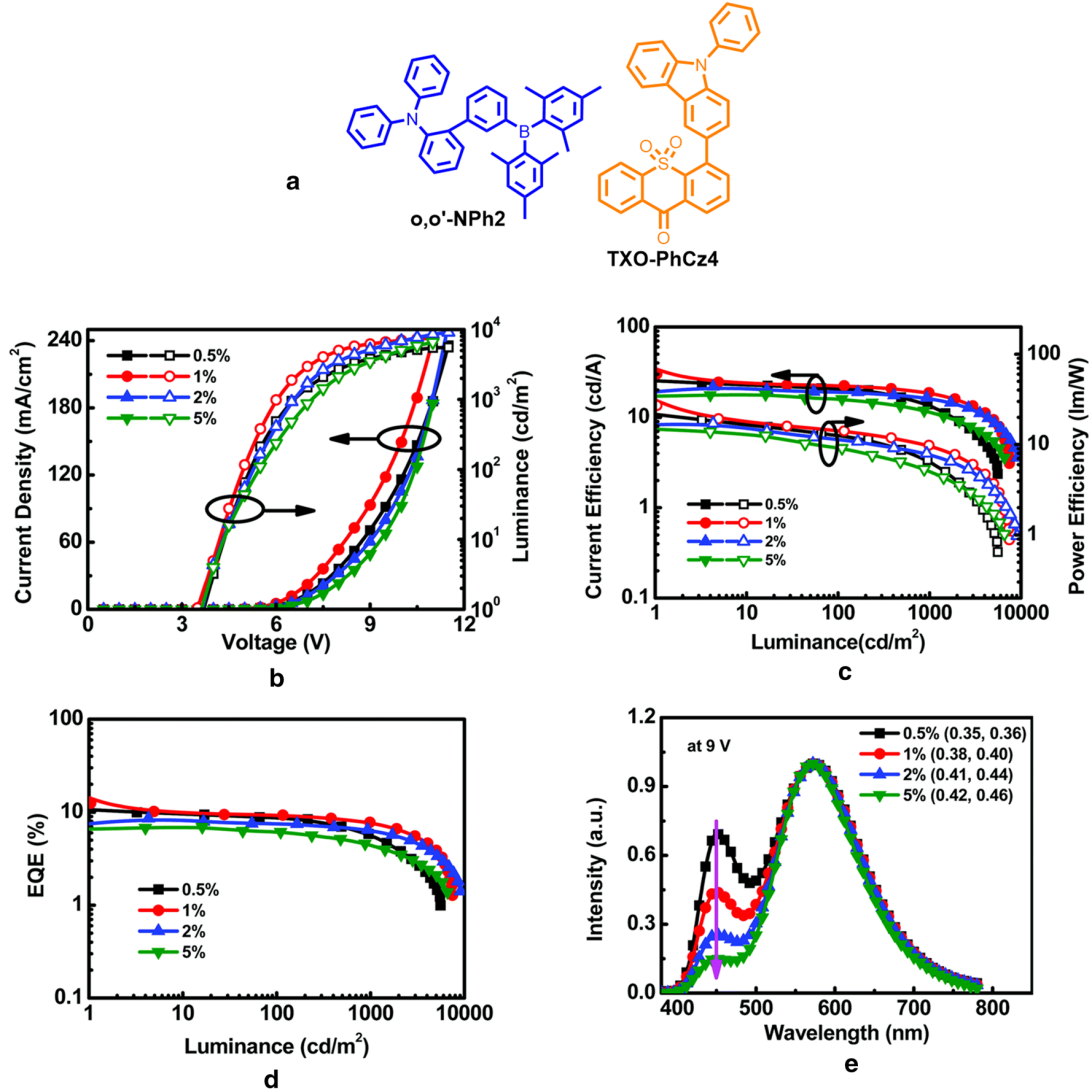
**a** Molecular structures of the emitters. The EL characteristics of WOLEDs fabricated with different dopant concentrations of TXO-PhCz4, **b** current density–voltage–luminance curves, **c** current efficiency and power efficiency versus luminance curves, **d** EQE-luminance curves, **e** EL spectra of all devices at 9 V

Any high performance WOLEDs comprises of a high FWHM and large spectral overlap among multi-component emitters. Wang and co-workers demonstrated a simple bi-component WOLED by adopting blue emitting TADF material, 20-(dimesitylboranyl)-*N*,*N*-diphenyl-[1,10-biphenyl]-2-amine (o,o′-NPh2), and yellow emitting TADF molecule 4-phenyl-40-carbazole-9-*H*-thioxanthen-9-one-10,10-dioxide (TXOPhCz4) as dopant (as shown in Fig. 19a) [50]. TXOPhCz4 dispersed in o,o′-NPh2 formed a bi-component EML for WLEDs (Fig. 19). The device architecture had ITO/HAT-CN (5 nm)/TAPC (30 nm)/mCP (15 nm)/x wt% TXOPhCz4:o,o′-NPh2 (20 nm)/TmPyPB (50 nm)/LiF (0.9 nm)/Al (90 nm), dopant concentration varied from 0.5 to 5.0 wt% and an optimized device holds best results among all bi-component WOLEDs with 12.5% maximum EQE, 30.2 cd/A current efficiency, and 27.1 lm/W power efficiency with (0.38, 0.40) CIE coordinates and CRI value of 77 at 9 V (Table 2).

**Fig. 20:**
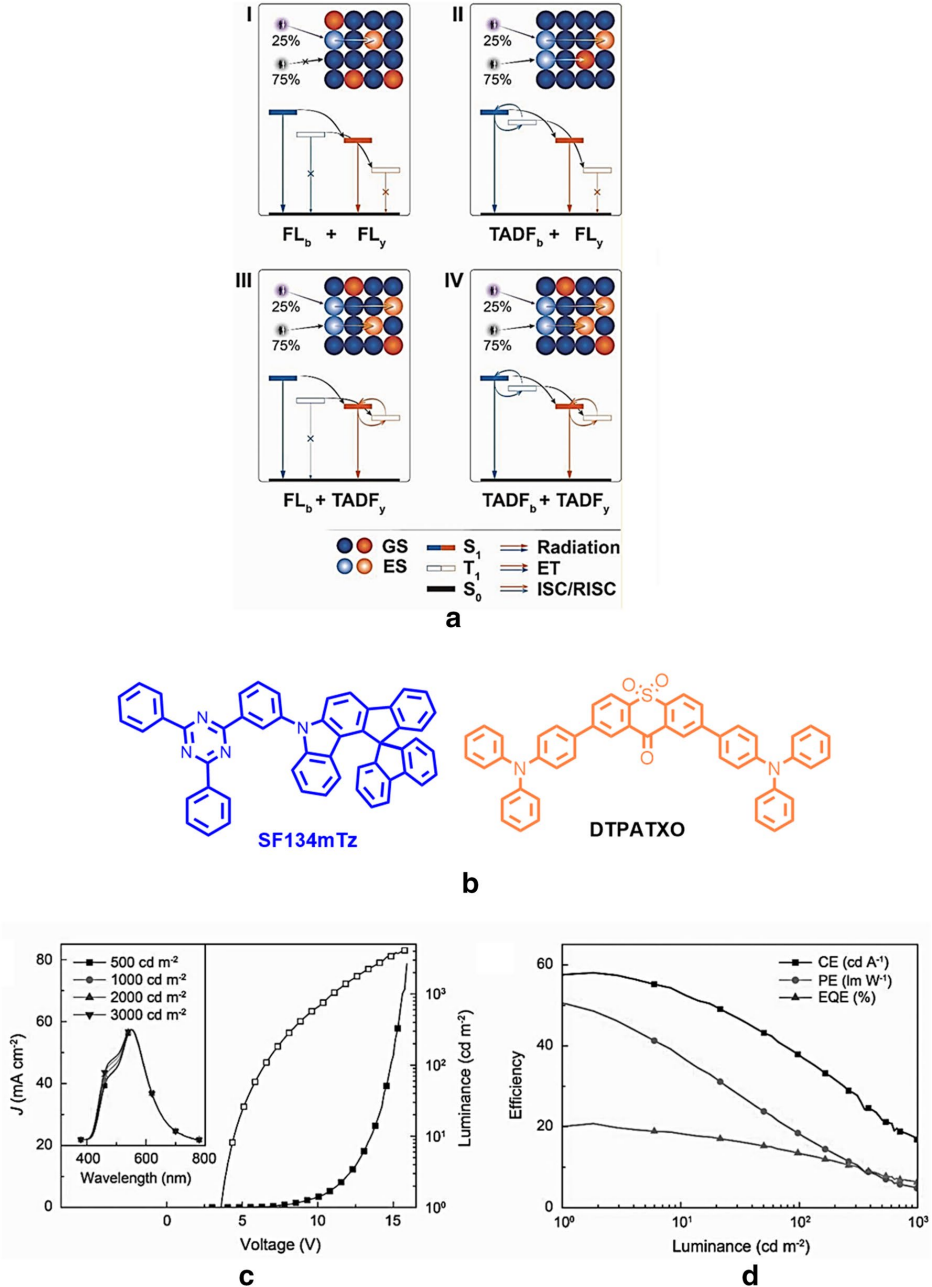
**a** Strategies for white light generation from the device. GS, ES, and ET, ground state, excited state and energy transfer respectively. **b** DTPATXO a yellow TADF structure, **c** luminance and J–V and EL spectra (inset) of the devices, **d** luminance efficiency curves of the devices (Reproduced with permission from Ref. [51] Copyright 2018, ©WILEY–VCH Verlag GmbH &amp; Co. KGaA, Weinheim)

Wang and co-workers established an edge-spiro strategy to develop an efficient blue TADF host, SFI34mTz for all-TADF WOLEDs (type IV), by considering the issue of interaction-induced quenching between adjacent TADF molecules and fluorescence molecules, and manifested previously explored F-WOLEDs (Type 1, II, and III) (shown in Fig. 20) [51]. A series of spiro[fluorene-indenocarbazole] (SFI)-based SFInxTz (n = 23 and 34; x = m and p), have been synthesized, SFI23mTz (5′-(3-(4,6-diphenyl-1,3 5-triazin-2-yl)phenyl)-5′*H*-spiro[fluorene-9,7′-indeno[2,1-b] carbazole]), SFI23pTz (5′-(4-(4,6-diphenyl-1,3,5-triazin-2-yl) phenyl)-5′*H*-spiro[fluorene-9,7′-indeno[2,1-b]carbazole]), SFI34mTz (5′-(3-(4,6-diphenyl-1,3,5-triazin-2-yl)phenyl)-5′*H*spiro[fluorene-9,12′-indeno[1,2-c]carbazole]), and SFI34pTz (5′-(4-(4,6-diphenyl-1,3,5-triazin-2-yl)phenyl)-5′*H*-spiro[fluorene-9,12′-indeno[1,2-c]carbazole]), these blue TADF dyes are ornamented with different spiro-fluorene groups to achieve a low ∆E_ST_ and high PLQY, Φ_PL_ due to a strong steric hindrance and good electronic distribution within the molecule. However, among all the designed emitters SFI34pTz realized a true blue electroluminescence with 25.3% of maximum EQE with CIE coordinates (0.15, 0.20), revealing a potential host material to suppress intermolecular interaction. Furthermore, 2,7-bis(4-(diphenylamino) phenyl)-9*H*-thioxanthen-9-one 10,10-dioxide (DTPATXO), a new yellow TADF dye, has been used as dopant to configure a white TADF device with the architecture shown in Table 2. Significantly, the fabricated WOLED accompanied a turn on voltage as low as 3.6 V, with nearly unchanged CIE coordinates of (0.32 ± 0.01, 0.42 ± 0.02) at an enhanced luminance of 500 to 3000 cd/m^2^, CCT in the range of 4962–6038 K. These WOLEDs resulted in EQE values of 22.9%, current efficiency of 58.0 cd/A and power efficiency 52.4 lm/W.

### Single-layer TADF-WOLEDs

**Fig. 21:**
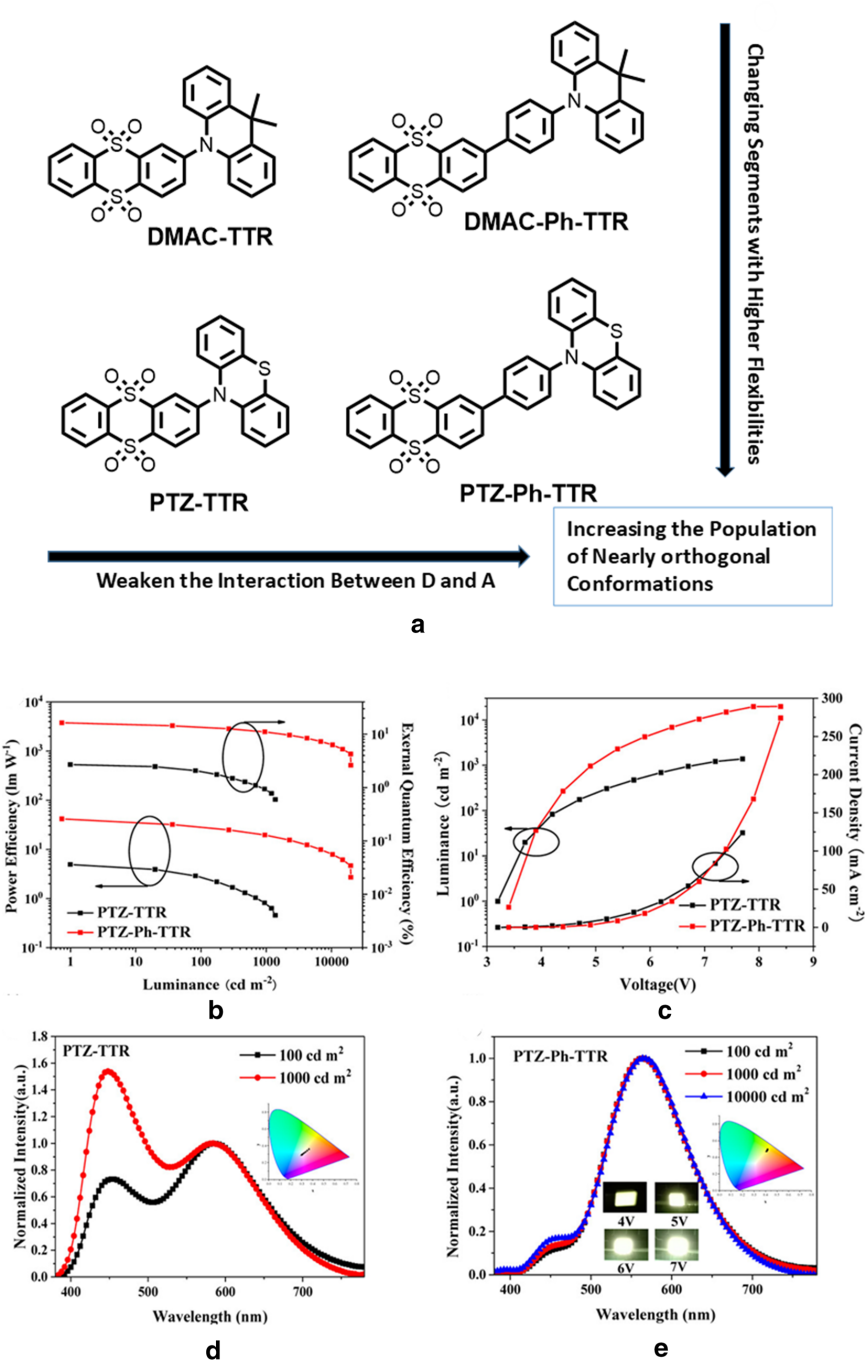
**a** Molecular structures of the emitters and strategical relationships. **b** PEluminance-EQE curves. **c** J–V and luminescence curves and normalized EL spectra of **d** PTZ-TTR (inset: CIE variations from 100 to 1000 cd/m^2^) and **e** PTZ-Ph-TTR (inset: CIE variations at different voltage) (Reproduced with permission from Ref. [52] Copyright © 2018, American Chemical Society)

WOLEDs based on multi-EML or multi-dopant single-EML device architecture has certain complication with high device cost and reproducibility. Therefore, development of single layer white light emitting materials is a long time demand in high performances WOLED technology. Zhang and co-workers presented the concept of using two specific TADF emitters 2-(10*H*-phenothiazin-10-yl) thianthrene 5,5,10,10-tetraoxide (PTZ-TTR) and 2-(4-(10*H*-phenothiazin-10-yl)phenyl)thianthrene 5,5,10,10-tetraoxide (PTZ-Ph-TTR) and studied them with two previously described emitters 2-(9,9-dimethylacridin-10(9*H*)-yl)thianthrene 5,5,10,10-tetraoxide (DMACTTR) and 2-(4-(9,9-dimethylacridin-10(9*H*)-yl)phenyl)-thianthrene 5,5,10,10-tetraoxide (DMAC-Ph-TTR) [52]. These TADF emitters exhibited tunable planar and orthogonal dual stable conformations with two different emission and 100% excitons utilization realized (Fig. 21).

Very pure white light with CIE coordinates (0.33, 0.33) and high CRI value of 92 at 300 cd/m brightness have been achieved in PTZ-TTR-based WOLED. Whereas, nearly orthogonal PTZ-Ph-TTR based WOLED exhibited warm white light with CIE coordinate (0.41, 0.47) and a high maximum forward-viewing EQE of 16.34% (device configurations and results presented in Table 2).

**Fig. 22:**
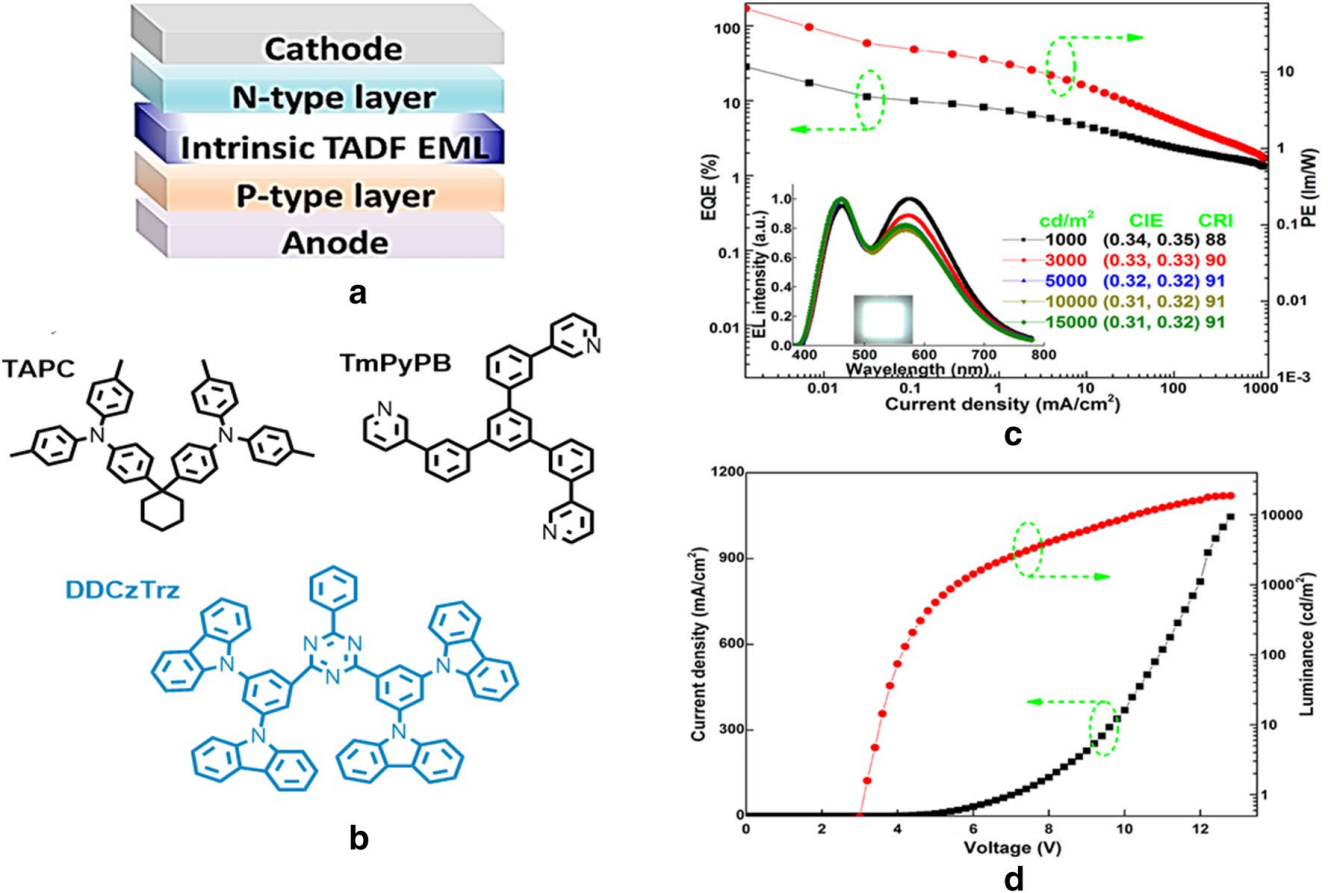
**a** Device configuration, **b** molecular structures of TAPC, TmPyPB, and DDCzTrz, **c** EQE and PE of W1. Inset: EL spectra at various luminances and an image of the WOLED, **d** current density and luminance of W1 (Reproduced with permission from Ref. [53] Copyright © 2018, American Chemical Society)

Recently, Luo et al. explored a very simple and high performing TADF WOLED, by introducing p-type (TAPC) and n-type (TmPyPB) layers, with a dopant-free single white light emitter (DDCzTrz) employed in this WOLED [53]. A high-quality white light emission with coordinates of (0.34, 0.35), brilliant CRI value of 91, a maximum total EQE, current efficiency (CE), and PE of 28.4%, 65.4 cd/A, and 68.5 lm/W at very low turn on voltage of 3.0 have been achieved from a superior p-i-n WOLED. The chemical structures and device characteristics are shown in Fig. 22. This simple device architecture and efficiencies reported a breakthrough record for WOLEDs and were analogous to the best reported doped TADF WOLEDs, with efficiencies comparable to the highest doping-free phosphorescent WOLEDs, including polymer and inorganic emitters.

## Perovskite white light emitting diodes

**Fig. 23:**
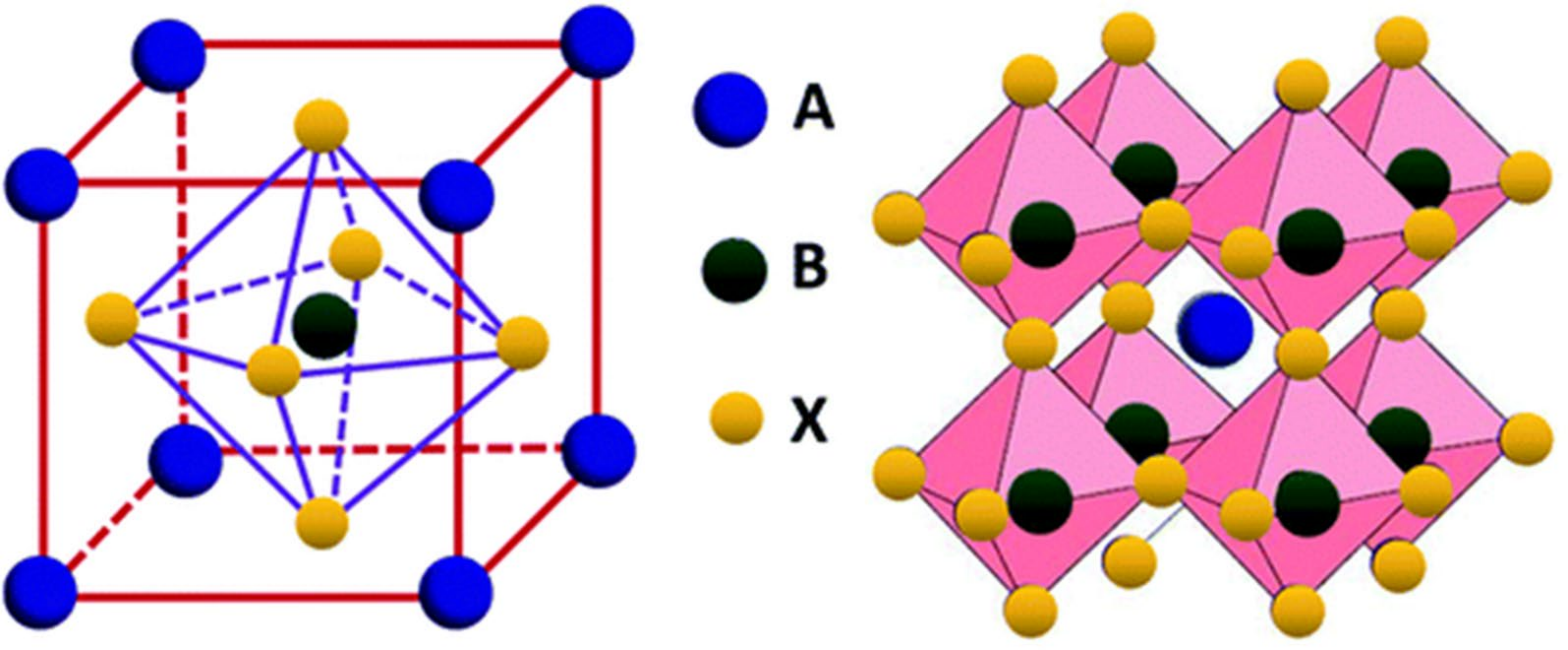
Perovskite structure ABX_3_ (Reproduced with permission from Ref. [67] Copyright © 2019, Royal Society of Chemistry)

The field of optoelectronics is recently witnessing the emergence of perovskite materials for various dynamic applications [54–56]. Record efficiency of 22.1% has been reported in the photovoltaics using these materials. The main reason for such high efficiency is the development of smooth films which are crystalline and defect-free. Moreover, such films can be formed using solution-processing technique. Another set of advantages that these materials possess are highly crystallinity, outstanding charge transport, easy way of grain size control, and surface trap density reduction by passivation with efficient carrier charge recombination which is the main requisite for application in white LEDs [57, 58]. Also, enhanced PLQY of with average values ca. 90% and 40% have been achieved in nanoparticles and thin films respectively [59–65]. Furthermore, the FWHM as narrow as 20 nm have also been reported and can be easily tunable covering the entire visible range [66]. A general perovskite structure that has general molecular formula ABX_3_ is shown in Fig. 23 where ‘A’ is an organic cation (i.e. CH_3_NH_3_^+^, CH_3_CH_2_NH_3_^+^), ‘B’ is metal cation (i.e. Pb^2+^, Sn^2+^) and ‘X’ is halogen anion (i.e. F^−^, Cl^−^, Br^−^, I^−^) [67].

**Fig. 24:**
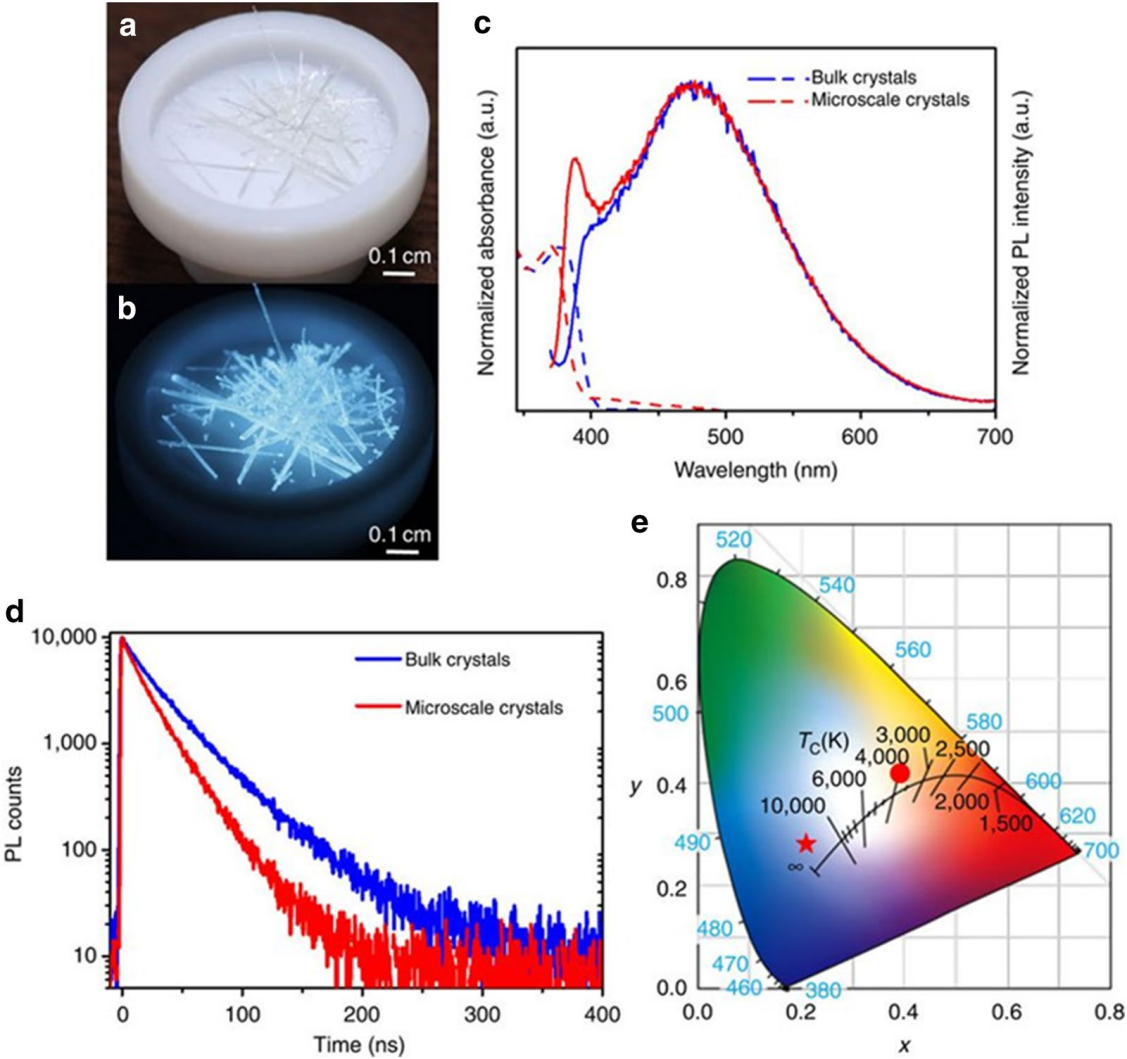
Perovskite crystals at **a** ambient temperature and **b** UV light, **c** absorbance spectra and emission spectra of the bulk and micro-scaled perovskite crystals at room temperature, **d** TRPL study bulk and micro-scaled perovskite crystals, **e** CIE color coordinates of the white perovskites (Reproduced with permission from Ref. [68] Copyright 2017, Nature Publishing Group)

Recently, both bulk and micro-crystals of 1D perovskites have been testified as white emitting materials. This work reports needle shaped, colourless crystals and exhibits strong bluish white emission under 365 nm UV irradiation. They report higher non-radiative decay rate for the microscale crystals compared to bulk crystals. The larger surface areas led to more defects that act as nonradiative decay paths. Further, for bulk and micro-crystals the reported PLQYs were 20% and 12% respectively which are the highest PLQY values (Fig. 24). The emission has λ_max_ at 475 nm and displays a broad spectrum covering the visible range. The CIE color coordinates for bulk and microscale crystals were found at (0.21, 0.27) and (0.21, 0.28) respectively [68].

Yet the most important concern remains the fabrication of stable and efficient devices emitting pure white light using perovskite materials. Numerous scientific and research groups have initiated the core reason behind this and the focus has now shifted to designing interlayers for increasing the stability by improving the interfaces. As of now, blue LEDs have also been accomplished using all-inorganic perovskite using perovskites. In 2015, this was demonstrated using CsPbX_3_ (X = Cl, Br, I) quantum dots (QDs) and these QDs were utilized in devices bearing architecture of ITO/PEDOT:PSS/PVK/QDs/TPBi/LiF/Al. Hot-injection method was used in the synthesis of QDs and their size and halide composition helped tuning of the luminescence wavelength. A luminance of 742 cd/m^2^ and an EQE of 0.07% were the output of the blue devices.

**Fig. 25:**
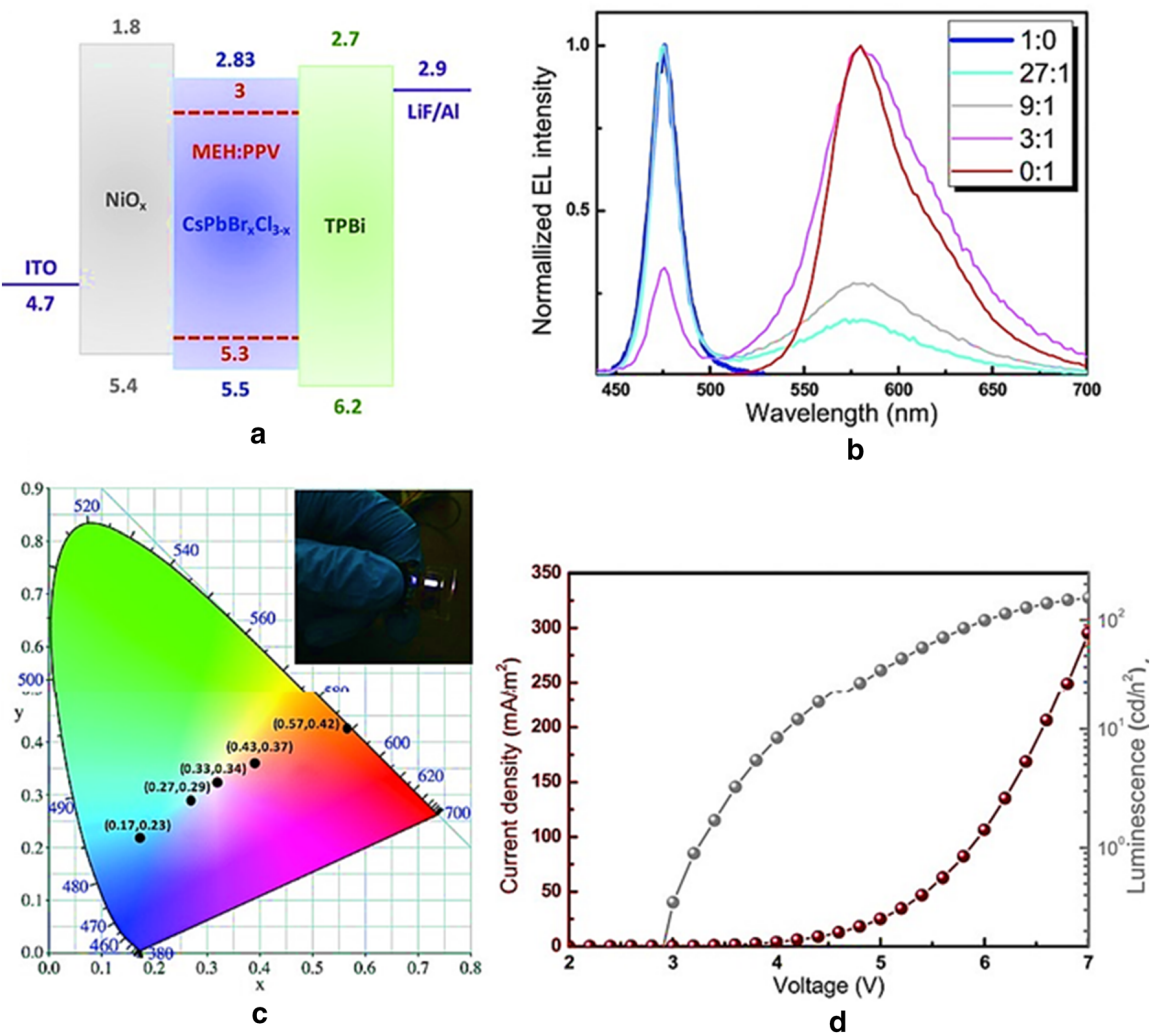
**a** White perovskite LED energy diagram, **b** EL spectra of MEH: PPV/perovskite active layer for different weight ratio, **c** the variation of the CIE color coordinates upon varying the weight ratio [inset: image of a working white PeLED], **d** the current density–luminance–voltage curve of white PeLEDs (Reproduced with permission from Ref. [69] Copyright 2017, ©WILEY–VCH Verlag GmbH & Co. KGaA, Weinheim)

By using the device architecture ITO/NiO_x_/CsPbBr_x_Cl_3−x_/TPBi/LiF:Al, the output parameters like brightness 350 cd/m^2^ and the current efficiency 0.18 cd/A has also been achieved. Apart from the blue light emitting diode (LED), the authors have also established a white LED with an active layer comprising a blend of orange emitting conjugated polymer i.e., poly[2-methoxy-5-(2-ethylhexyloxy)-1,4-phenylenevinylene] (MEH:PPV) and CsPbBrxCl_3−x_ QDs, using a similar device architecture (ITO/NiOx/CsPbBr_x_Cl_3−x_:MEH:PPV/TPBi/LiF:Al). Nickel oxide (NiO_x_) provides an enhanced thermal and chemical stability compared to PEDOT:PSS and it was used as a hole transporting layer also. TPBi is used as an electron transport/hole blocking layer since it has a strong hole blocking property compared with BPhen or BCP (Fig. 25). The white light emitting device had CIE coordinates of (0.33, 0.34) and a maximum brightness of ca. 100 cd/m^2^ for an applied voltage of 7 V [69].

**Fig. 26:**
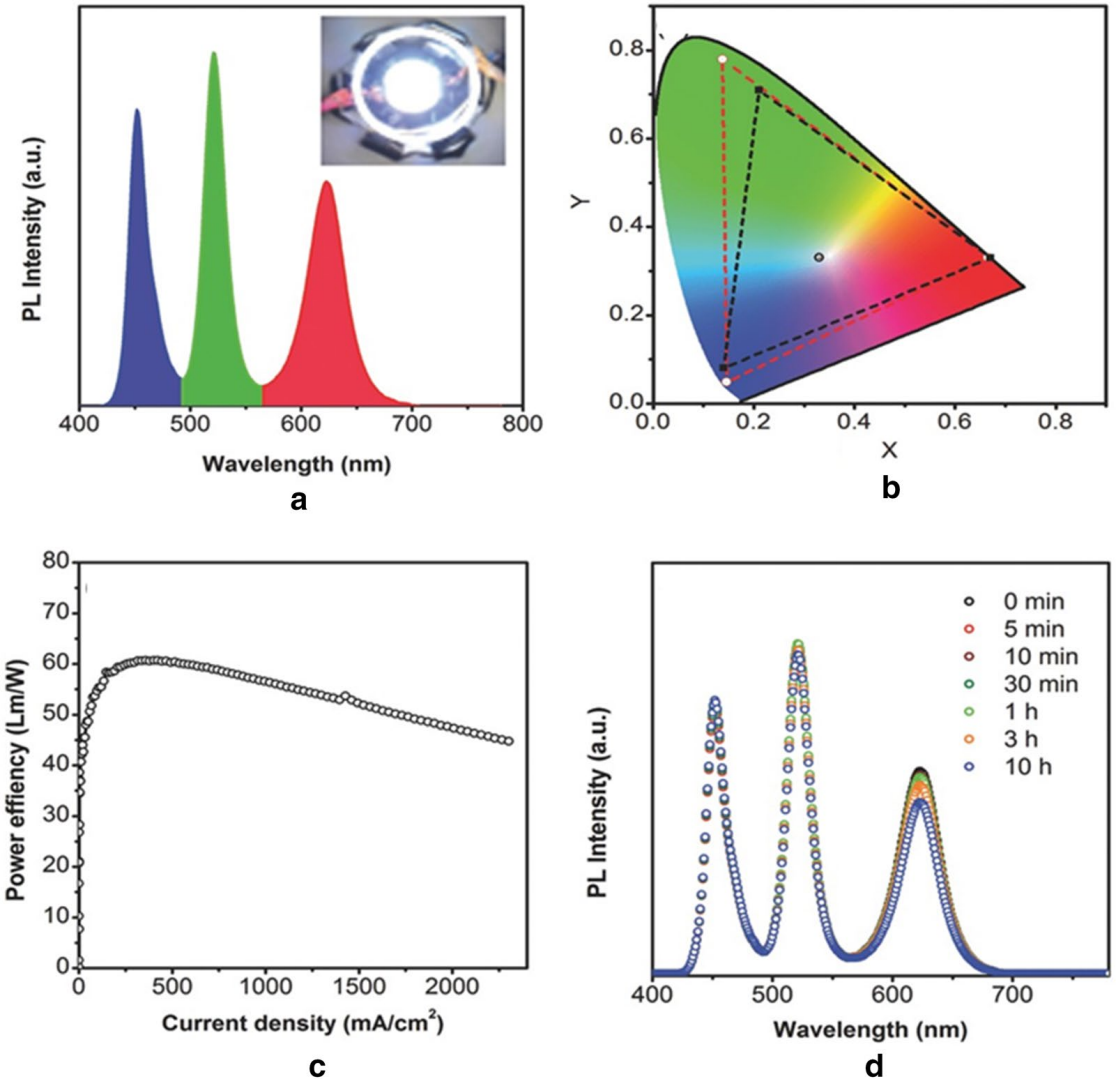
**a** PL spectra, **b** color triangle and the CIE color coordinates of the WLED device, **c** power efficiency vs. current density spectrum of the WLED. **d** PL spectra of the WLED measured at different time (Reproduced with permission from Ref. [70] Copyright 2016, ©WILEY–VCH Verlag GmbH & Co. KGaA, Weinheim)

Li et al. in 2016, reported color tunable red-white, green-white, and warm-white LEDs on varying the mass ratios of the green and red perovskites (Fig. 26). The authors used recrystallization technique to synthesize quantum dots (QDs) of CsPbX_3_ (X = Cl, Br, and I) which are also highly emissive. These QDs presented PLQYs of 70%, 95%, and 80% with a FWHMs of 18, 20, and 35 nm for CsPbClBr_2_ (λ_max_ = 478 nm), CsPbBr_3_ (λ_max_ = 513 nm) and CsPbBr_1.2_I_1.8_ (λ_max_ = 628 nm) respectively. The trapping of the excitons are prevented by using a halogen-rich surface, thereby giving very high PLQY values. PMMA is solubilized in chloroform and the green and red QDs were dispersed into the mixture to form QDs/PMMA layers which are mounted on a blue emitting backlight. In this system a green/red mass ratio of 1:5 provides diodes with CIE color coordinates of (0.33, 0.30) and a CCT of ca. 6000 K [70].

## Conclusions

In the present review, an excellent association of old and modern concept of cost-effective materials and device structure for white lighting technology applications has been summarized and extrapolated. In particular, this article demonstrated and focused on design, and development of novel synthesis strategy, mechanistic insights and device engineering for solution processed low cost WOLEDs device. By tuning chromaticity adjustable emissive molecular structural unit either by intramolecular, intermolecular fashion in single layer or by multilayer structural perturbations, doping with foreign additives in emissive layer, high performance device efficiencies can be achieved. Herein, an overview of the existing routes towards white lighting devices and corresponding materials used, including polymer white light emitting diode (PWLED), small molecules based thermally activated delayed fluorescence (TADF), emitters, perovskite materials based thin film light-emitting diodes (PeLEDs) and hybrid LEDs (HLEDs), color down-converting coatings with corresponding best efficiencies ever realized have also been summarized. Overall, it is expected that this review will deliver information on the wide-ranging development in this area and provide an overview of the current state in the field of white SSL encompassing various materials and device architecture choices, provide directions for new innovations in many of these aspects as this research topic matures over the next few years to an all-purpose lighting and display technology for the future applications.

## Data Availability

Wherever necessary, relevant citations and copyright has been obtained for data and materials presented herein.
